# Identification of causal effects in case-control studies

**DOI:** 10.1186/s12874-021-01484-7

**Published:** 2022-01-07

**Authors:** Bas B. L. Penning de Vries, Rolf H. H. Groenwold

**Affiliations:** 1 Department of Clinical Epidemiology, Leiden University Medical Center, Leiden, PO Box 9600, 2300 RC The Netherlands; 2 Department of Biomedical Data Sciences, Leiden University Medical Center, Leiden, The Netherlands

**Keywords:** Causal inference, Case-control designs, Identifiability

## Abstract

**Background:**

Case-control designs are an important yet commonly misunderstood tool in the epidemiologist’s arsenal for causal inference. We reconsider classical concepts, assumptions and principles and explore when the results of case-control studies can be endowed a causal interpretation.

**Results:**

We establish how, and under which conditions, various causal estimands relating to intention-to-treat or per-protocol effects can be identified based on the data that are collected under popular sampling schemes (case-base, survivor, and risk-set sampling, with or without matching). We present a concise summary of our identification results that link the estimands to the (distribution of the) available data and articulate under which conditions these links hold.

**Conclusion:**

The modern epidemiologist’s arsenal for causal inference is well-suited to make transparent for case-control designs what assumptions are necessary or sufficient to endow the respective study results with a causal interpretation and, in turn, help resolve or prevent misunderstanding. Our approach may inform future research on different estimands, other variations of the case-control design or settings with additional complexities.

**Supplementary Information:**

The online version contains supplementary material available at (10.1186/s12874-021-01484-7).

## Introduction

In causal inference, it is important that the causal question of interest is unambiguously articulated [1]. The causal question should dictate, and therefore be at the start of, investigation. When the target causal quantity, the estimand, is made explicit, one can start to question how it relates to the available data distribution and, as such, form a basis for estimation with finite samples from this distribution.

The counterfactual framework offers a language rich enough to articulate a wide variety of causal claims that can be expressed as what-if statements [1]. Another, albeit closely related, approach to causal inference is target trial emulation, an explicit effort to mitigate departures from a study (the ‘target trial’) that, if carried out, would enable one to readily answer the causal what-if question of interest [2]. While it may be too impractical or unethical to implement, making explicit what a target trial looks like has particular value in communicating the inferential goal and offers a reference against which to compare studies that have been or are to be conducted.

The counterfactual framework and emulation approach have become increasingly popular in observational cohort studies. Case-control studies, however, have not yet enjoyed this trend. A notable exception is given by Dickerman et al. [3], who recently outlined an application of trial emulation with case-control designs to statin use and colorectal cancer.

In this paper, we give an overview of how observational data obtained with case-control designs can be used to identify a number of causal estimands and, in doing so, recast historical case-control concepts, assumptions and principles in a modern and formal framework.

## Preliminaries

### Identification versus estimation

An estimand is said to be identifiable if the distribution of the available data is compatible with exactly one value of the estimand, or therefore, if the estimand can be expressed as a functional of the available data distribution. Identifiability is a relative notion as it depends on which data are available as well as on the assumptions one is willing to make. Identification forms a basis for estimation with finite samples from the available data distribution [4]. Once the estimand has been made explicit and an identifying functional established, estimation is a purely statistical problem. While the identifying functional will often naturally translate into a plug-in estimator, there is, however, generally more than one way to translate an identifiability result into an estimator and different estimators may have important differences in their statistical properties. Moreover, while the estimand may be identifiable, there need not exist an estimator with the desired properties (see e.g. [5]). Here, our focus is on identification, so that the purely statistical issues of the next step in causal inference, estimation, can be momentarily put aside.

### Case-control study nested in cohort study

To facilitate understanding, it is useful to consider every case-control study as being “nested” within a cohort study. A case-control study could be considered as a cohort study with missingness governed by the control sampling scheme. Therefore, when the observed data distribution of a case-control study is compatible with exactly one value of a given estimand, then so is the available or observed data distribution of the underlying cohort study. In other words, identifiability of an estimand with a case-control study implies identifiability of the estimand with the cohort study within which it is nested (conceptually). The converse is not evident and in fact may not be true. In this paper, the focus is on sets of conditions or assumptions that are sufficient for identifiability in case-control studies.

### Set-up of underlying cohort study

**Fig. 1:**
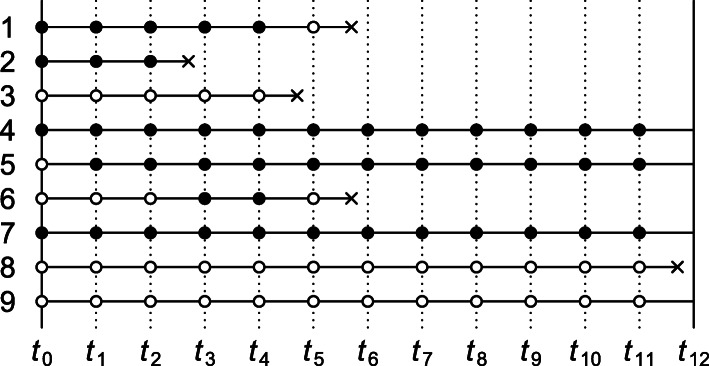
Illustration of possible courses of follow-up of an individual for a study with baseline *t*_0_ and administrative study end *t*_12_. Solid bullets indicate ‘exposed’; empty bullets indicate ‘not exposed’. The incident event of interest is represented by a cross

Consider a time-varying exposure *A*_*k*_ that can take one of two levels, 0 or 1, at *K* successive time points *t*_*k*_ (*k*=0,1,...,*K*−1), where *t*_0_ denotes baseline (cohort entry or time zero). Study participants are followed over time until they sustain the event of interest or the administrative study end *t*_*K*_, whichever comes first. We denote by *T* the time elapsed from baseline until the event of interest and let *Y*_*k*_=*I*(*T*<*t*_*k*_) indicate whether the event has occurred by *t*_*k*_. The lengths between the time points are typically fixed at a constant (e.g., of one day, week, or month). Figure 1 depicts twelve equally spaced time points over, say, twelve months with several possible courses of follow-up of an individual. As the figure illustrates, individuals can switch between exposure levels during follow-up, as in any truly observational study. Apart from exposure and outcome data, we also consider a (vector of) covariate(s) *L*_*k*_, which describes time-fixed individual characteristics or time-varying characteristics typically relating to a time window just before exposure or non-exposure at *t*_*k*_,*k*=0,1,...,*K*−1.

### Causal contrasts

Although there are many possible contrasts, particularly with time-varying exposures, for simplicity we consider only two pairs of mutually exclusive interventions: (1) setting baseline exposure *A*_0_ to 1 versus 0; and (2) setting all of *A*_0_,*A*_1_,...,*A*_*K*−1_ to 1 (‘always exposed’) versus all to 0 (‘never exposed’). For *a*=0,1, we let counterfactual outcome *Y*_*k*_(*a*) indicate whether the event has occurred by *t*_*k*_ under the baseline-only intervention that sets *A*_0_ to *a*. By convention, we write $\overline {1}=(1,1,...,1)$ and $\overline {0}=(0,0,...,0)$, and let $Y_{k}(\overline {1})$ and $Y_{k}(\overline {0})$ indicate whether the event has occurred by *t*_*k*_ under the intervention that sets all elements of (*A*_0_,*A*_1_,...,*A*_*K*−1_) to 1 and all to 0, respectively. Further details about the notation and set-up are given in Supplementary Appendix A.

### Case-control sampling

The fact that each time-specific exposure variable can take only one value per time point means that at most one counterfactual outcome can be observed per individual. This type of missingness is common to all studies. Relative to the cohort studies within which they are nested, case-control studies have additional missingness, which is governed by the control sampling scheme. In this paper, we focus on three well-known sampling schemes: case-base sampling, survivor sampling, and risk-set sampling. The next sections give an overview of conditions under which intention-to-treat and always-versus-never-exposed per-protocol effects can be identified with the data that are observed under these sampling schemes.

## Case-control studies without matching

Table 1 summarises a number of identification results for case-control studies without matching. Each result consists of one of the three aforementioned sampling schemes, an estimand, a set of assumptions, and an identification strategy. Under the conditions of the “Sampling scheme” and “Assumptions” columns, an identifying functional of the estimand of the “Estimand” column is obtained by following the steps of the “Identification strategy” column. More formal statements and proofs are given in Supplementary Appendix B.

**Table 1:** Overview of (non-parametric) identification results for case-control studies without matching

Sampling scheme	Estimand	Assumptions	Identification strategy
Case-base	Risk ratio for intention-to-treat effect $\frac {\Pr (Y_{K}(1)=1)}{\Pr (Y_{K}(0)=1)}$	∙Control selection *S* independent of baseline covariates *L*_0_ and exposure *A*_0_ ∙Consistency ∙Baseline exchangeability given *L*_0_ ∙Positivity (Theorem 1, Supplementary Appendix B)	1. Derive time-fixed IP weights *W* from control data 2. Compute the baseline exposure odds among cases, weighted by *W* 3. Compute the baseline exposure odds among controls, weighted by *W* 4. Take the ratio of the results of steps 2 and 3
Survivor	Odds ratio for intention-to-treat effect $\frac {\text {Odds}(Y_{K}(1)=1|L_{0})}{\text {Odds}(Y_{K}(0)=1|L_{0})}$	∙Control selection *S* independent of baseline exposure *A*_0_ given baseline covariates *L*_0_ and survival until *t*_*K*_ (*Y*_*K*_=0) ∙Consistency ∙Baseline exchangeability given *L*_0_ ∙Positivity (Theorem 3, Supplementary Appendix B)	1. Derive the conditional baseline exposure odds given *L*_0_ among cases 2. Derive the conditional baseline exposure odds given *L*_0_ among controls 3. Take the ratio of the results of steps 1 and 2
Risk-set	Hazard ratio for intention-to-treat effect $\frac {\Pr (Y_{k+1}(1)=1|Y_{k}(1)=0)}{\Pr (Y_{k+1}(0)=1|Y_{k}(0)=0)}$	∙Control selection *S*_*k*_ independent of baseline covariates *L*_0_ and exposure *A*_0_ given eligibility at *t*_*k*_ (*Y*_*k*_=0) with constant sampling probability among those eligible ^*†*^ ∙Consistency ∙Baseline exchangeability given *L*_0_ ∙Positivity ∙Constant counterfactual hazards (Theorem 4, Supplementary Appendix B)	1. Derive time-fixed IP weights *W* from control data 2. Compute baseline exposure odds among cases, weighted by *W* 3. Compute baseline exposure odds among controls, weighted by *W* times $\sum _{k=0}^{K-1}S_{k}$, the number of times selected as a control 4. Take the ratio of the results of steps 2 and 3
	Hazard ratio for per-protocol effect $\frac {\Pr (Y_{k+1}(\overline {1})=1|Y_{k}(\overline {1})=0)}{\Pr (Y_{k+1}(\overline {0})=1|Y_{k}(\overline {0})=0)}$	∙Control selection *S*_*k*_ independent of covariate and exposure history up to *t*_*k*_ given eligibility at *t*_*k*_ (*Y*_*k*_=0) with constant sampling probability among those eligible ^*†*^ ∙Consistency ∙Sequential conditional exchangeability ∙Positivity ∙Constant counterfactual hazards (Theorem 6, Supplementary Appendix B)	1. Derive time-varying IP weights *W*_*k*_ from control data 2. Censor from time of protocol deviation 3. Compute (baseline) exposure odds among cases, weighted by those weights *W*_*k*_ such that *Y*_*k*_=0 and *Y*_*k*+1_=1 4. Compute (baseline) exposure odds among all controls, weighted by $\sum _{k=0}^{K-1}W_{k}S_{k}$, the weighted number of times selected as a control 5. Take the ratio of the results of steps 3 and 4

See text or Supplementary material for elaboration on assumptions. ^*†*^Weaker/alternative control selection assumptions are given in the Supplementary material

In all case-control studies that we consider in this section, cases are compared with controls with regard to their exposure status via an odds ratio, even when an effect measure other than the odds ratio is targeted. An individual qualifies as a case if and only if they sustain the event of interest by the administrative study end (i.e., *Y*_*K*_=1) and adhered to one of the protocols of interest until the time of the incident event. In Fig. 1, the individual represented by row 1 is therefore regarded as a case (an exposed case in particular) in our investigation of intention-to-treat effects but not in that of per-protocol effects. Whether an individual (also) serves as a control depends on the control sampling scheme.

### Case-base sampling

The first result in Table 1 describes how to identify the intention-to-treat effect as quantified by the marginal risk ratio

$$\begin{array}{*{20}l} \frac{\Pr(Y_{K}(1)=1)}{\Pr(Y_{K}(0)=1)} \end{array} $$

under case-base sampling. (For identification of a conditional risk ratio, see Theorem 2 of Supplementary Appendix B.) Case-base sampling, also known as case-cohort sampling, means that no individual who is at risk at baseline of sustaining the event of interest is precluded from selection as a control. Selection as a control, *S*, is further assumed independent of baseline covariate *L*_0_ and exposure *A*_0_. Selecting controls from survivors only (e.g., rows 4, 5, 7 and 9 in Fig. 1) violates this assumption when survival depends on *L*_0_ or *A*_0_.

To account for baseline confounding, inverse probability weights could be derived from control data according to

1 $$\begin{array}{*{20}l}  W &= \frac{A_{0}}{\Pr(A_{0}=1|L_{0},S=1)}+\frac{1-A_{0}}{1-\Pr(A_{0}=1|L_{0},S=1)}. \end{array} $$

We then compute the odds of baseline exposure among cases and among controls in the pseudopopulation that is obtained by weighting everyone by subject-specific values of *W*. The ratio of these odds coincides with the target risk ratio under the three key identifiability conditions of consistency, baseline conditional exchangeability and positivity [1]. Consistency here means that for *a*=0,1,*Y*_*K*_(*a*)=*Y*_*K*_ if *A*_0_=*a*, baseline conditional exchangeability that for *a*=0,1,*A*_0_ is independent of *Y*_*K*_(*a*), and positivity that 0< Pr(*A*_0_=1|*L*_0_,*S*=1)<1.

The identification result for case-base sampling suggests a plug-in estimator: replace all functionals of the theoretical data distribution with sample analogues. For example, to obtain the weight for an individual with baseline covariate level *l*_0_, replace the theoretical propensity score Pr(*A*_0_=1|*L*_0_=*l*_0_,*S*=1) with an estimate $\widehat {\Pr }(A_{0}=1|L_{0}=l_{0},S=1)$ derived from a fitted model (e.g., a logistic regression model) that imposes parametric constraints on the distribution of *A*_0_ given *L*_0_ among the controls.

### Survivor sampling

With survivor (cumulative incidence or exclusive) sampling, a subject is eligible for selection as a control only if they reach the administrative study end event-free. To identify the conditional odds ratio of baseline exposure versus baseline non-exposure given *L*_0_,

$$\begin{array}{*{20}l} \frac{\text{Odds}(Y_{K}(1)=1|L_{0})}{\text{Odds}(Y_{K}(0)=1|L_{0})}, \end{array} $$

selection as a control, *S*, is assumed independent of baseline exposure *A*_0_ given *L*_0_ and survival until the end of study (i.e., *Y*_*K*_=0).

As is shown in Supplementary Appendix B, Theorem 3, the above odds ratio is identified by the ratio of the baseline exposure odds given *L*_0_ among the cases versus controls, provided the key identifiability conditions of consistency, baseline conditional exchangeability, and positivity are met.

All estimands in Table 1 describe a marginal effect, except for the odds ratio, which is conditional on baseline covariates *L*_0_. The corresponding marginal odds ratio

$$\begin{array}{*{20}l} \frac{\text{Odds}(Y_{K}(1)=1)}{\text{Odds}(Y_{K}(0)=1)} \end{array} $$

is not identifiable from the available data distribution under the stated assumptions (see remark to Theorem 3, Supplementary Appendix B). However, approximate identifiability can be achieved by invoking the rare event assumption (or rare disease assumption), in which case the marginal odds ratio approximates the marginal risk ratio.

### Risk-set sampling for intention-to-treat effect

With risk-set (or incidence density) sampling, for all time windows [*t*_*k*_,*t*_*k*+1_),*k*=0,...,*K*−1, every subject who is event-free at *t*_*k*_ is eligible for selection as a control for the period [*t*_*k*_,*t*_*k*+1_). This means that study participants may be selected as a control more than once.

Consider the intention-to-treat effect quantified by the marginal (discrete-time) hazard ratio (or rate ratio)

$$\begin{array}{*{20}l} \frac{\Pr(Y_{k+1}(1)=1|Y_{k}(1)=0)}{\Pr(Y_{k+1}(0)=1|Y_{k}(0)=0)}. \end{array} $$

(For identification of a conditional hazard ratio, see Theorem 5, Supplementary Appendix B.) For identification of the above marginal hazard ratio under risk-set sampling, it is assumed that selection as a control between *t*_*k*_ and *t*_*k*+1_,*S*_*k*_, is independent of the baseline covariates and exposure given eligibility at *t*_*k*_ (i.e., *Y*_*k*_=0). It is also assumed that the sampling probability among those eligible, Pr(*S*_*k*_=1|*Y*_*k*_=0), is constant across time windows *k*=0,...,*K*−1. To this end, it suffices that the marginal hazard Pr(*Y*_*k*+1_=1|*Y*_*k*_=0) remains constant across time windows and that every *k*th sampling fraction Pr(*S*_*k*_=1) is equal, up to a proportionality constant, to the probability Pr(*Y*_*k*+1_=1,*Y*_*k*_=0) of an incident case in the *k*th window (see remark to Theorem 4, Supplementary Appendix B). For practical purposes, this suggests sampling a fixed number of controls for every case from among the set of eligible individuals. To illustrate, consider Fig. 1 and note first of all that the individual represented by row 1 trivially qualifies as a case, because the individual survived until the event occurred. Because the event was sustained between *t*_5_ and *t*_6_, the proposed sampling suggests selecting a fixed number of controls from among those who are eligible at *t*_5_. Thus, rows (and only rows) 4 through 9 as well as row 1 itself in Fig. 1 qualify for selection as a control for this case. Even though the individual of row 1 is a case, the individual may also be selected as a control when the individuals of row 2, 3 and 6 (but not 8) sustain the event.

Once cases and controls are selected, we can start to derive inverse probability weights *W* according to Eq. 1 with *S* replaced with *S*_0_. We then compute the odds of baseline exposure among cases in the pseudopopulation that is obtained by weighting everyone by *W* and the odds of baseline exposure among controls weighted by *W* multiplied by the number of times the individual was selected as a control. The ratio of these odds coincides with the target hazard ratio under the three key identifiability conditions of consistency, baseline conditional exchangeability and positivity together with the assumption that the hazards in the numerator and denominator of the causal hazard ratio are constant across the time windows.

The consistency and exchangeability conditions are here slightly stronger than those of the previous subsections. Specifically, Theorem 4 (Supplementary Appendix B) requires consistency of the form: for all *k*=1,...,*K* and *a*=0,1,*Y*_*k*_(*a*)=*Y*_*k*_ if *A*_0_=*a*. The exchangeability condition requires, for *a*=0,1, that conditional on *L*_0_, the counterfactual outcomes *Y*_1_(*a*),...,*Y*_*K*_(*a*) are jointly independent of *A*_0_. The positivity condition takes the same form as in the previous subsections (i.e., 0< Pr(*A*_0_=*a*|*L*_0_,*S*_0_=1)<1).

### Risk-set sampling for per-protocol effect

For the per-protocol effect quantified by the (discrete-time) hazard ratio (or rate ratio)

$$\begin{array}{*{20}l} \frac{\Pr(Y_{k+1}(\overline{1})=1|Y_{k}(\overline{1})=0)}{\Pr(Y_{j+1}(\overline{0})=1|Y_{k}(\overline{0})=0)}, \end{array} $$

eligibility for selection as a control for the period [*t*_*k*_,*t*_*k*+1_) again requires that the respective subject is event-free at *t*_*k*_ (i.e., *Y*_*k*_=0). Selection as a control between *t*_*k*_ and *t*_*k*+1_,*S*_*k*_, is further assumed independent of covariate and exposure history up to *t*_*k*_ given eligibility at *t*_*k*_ (but see Supplementary Appendix B for a slightly weaker assumption). As for the intention-to-treat effect, it is also assumed that the probability to be selected as a control *S*_*k*_ given eligibility is constant across time windows. This assumption is guaranteed to hold if the marginal hazard Pr(*Y*_*k*+1_=1|*Y*_*k*_=0) remains constant across time windows and that every *k*th sampling fraction Pr(*S*_*k*_=1) is equal, up to a proportionality constant, to the probability of an incident case in the *k*th window. Figure 1 shows five incident events yet only three qualify as a case (rows 2, 3 and 8) when it concerns per-protocol effects. When the first case emerges (row 2), all rows meet the eligibility criterion for selection as a control. When the second emerges, the individual of row 2, who fails to survive event-free until *t*_4_, is precluded as a control. When the case of row 8 emerges, only the individuals of rows 4, 5, 7 and 9 are eligible as controls.

Once cases and controls are selected, we can start to derive time-varying inverse probability weights according to

$$\begin{array}{*{20}l} W_{k}&=\prod_{j=0}^{k}\left[\frac{A_{j}}{\Pr(A_{j}=1|L_{0},...,L_{j},A_{0},...,A_{j-1},Y_{j}=0,S_{j}=1)}\right.\\&\quad\left.+\frac{1-A_{j}}{1\,-\,\Pr(A_{j}\,=\,1|L_{0},...,L_{j},A_{0},...,A_{j-1},Y_{j}\,=\,0,S_{j}\,=\,1)\!}\right]. \end{array} $$

It is important to note that the weights are derived from control information but are nonetheless used to weight both cases and controls [6]. The denominators of the weights describe the propensity to switch exposure level. However, once the weights are derived, every subject is censored from the time that they fail to adhere to one of the protocols of interest for all downstream analysis. The uncensored exposure levels are therefore constant over time. We then compute the baseline exposure odds among cases, weighted by the weights *W*_*k*_ corresponding to the interval [*t*_*k*_,*t*_*k*+1_) of the incident event (i.e., *Y*_*k*_=0,*Y*_*k*+1_=1), as well as the baseline exposure odds among controls, weighted by $\sum _{k=0}^{K-1}W_{k}S_{k}$, the weighted number of times selected as control. The ratio of these odds equals the target hazard ratio under the three key identifiability conditions of consistency, sequential conditional exchangeability, and positivity together with the assumption that hazards in the numerator and denominator of the causal hazard ratio for the per-protocol effect are constant across the time windows. The consistency, exchangeability and positivity conditions take a somewhat different (stronger) form than in the previous subsections; we refer the reader to Supplementary Appendix A for further details.

## Case-control studies with matching

Table 2 gives an overview of identification results for case-control studies with exact pair matching. Formal statements and proofs are given in Supplementary Appendix C, which also includes a generalisation of the results of Table 2 to exact 1-to-*M* matching. While the focus in this section is on exact covariate matching, for partial matching we refer the reader to Supplementary Appendix D, where we consider parametric identification by way of conditional logistic regression.

**Table 2:** Overview of (non-parametric) identification results for case-control studies with exact pair matching

Sampling scheme	Estimand	Assumptions	Identification strategy
Case-base	Risk ratio for intention-to-treat effect $\frac {\Pr (Y_{K}(1)=1)}{\Pr (Y_{K}(0)=1)}$	∙Matched control exposure *A*^′^ sampled from the baseline exposure levels of all subjects with same baseline covariate level *L*_0_ as case, independently of the subjects’ baseline exposure or survival status ∙Consistency ∙Baseline conditional exchangeability ∙Positivity ∙Pr(*Y*_*K*_=1|*L*_0_=*l,A*_0_=1)/Pr(*Y*_*K*_=1|*L*_0_=*l,A*_0_=0) constant across levels *l* (Theorem 7, Supplementary Appendix C)	1. Compute the frequency of discordant case-control pairs with *A*_0_=1 and *A*^′^=02. Compute the frequency of discordant case-control pairs with *A*_0_=0 and *A*^′^=1 3. Take the ratio of the results of steps 1 and 2
Survivor	Odds ratio for intention-to-treat effect $\frac {\text {Odds}(Y_{K}(1)=1|L_{0})}{\text {Odds}(Y_{K}(0)=1|L_{0})}$	∙Matched control exposure *A*^′^ sampled from all the baseline exposure levels of all survivors (*Y*_*K*_=0) with same value for *L*_0_ as case, independently of the subjects’ baseline exposure ∙Consistency ∙Baseline conditional exchangeability ∙Positivity ∙Odds(*Y*_*K*_=1|*L*_0_,*A*_0_=1)/Odds(*Y*_*K*_=1|*L*_0_,*A*_0_=0) constant across levels *l* (Theorem 8, Supplementary Appendix C)	(Same as identification strategy for case-base sampling)
Risk-set	Hazard ratio for intention-to-treat effect $\frac {\Pr (Y_{k+1}(1)=1|L_{0},Y_{k}(1)=0)}{\Pr (Y_{k+1}(0)=1|L_{0},Y_{k}(0)=0)}$	∙For a case with incident event in [*t*_*k*_,*t*_*k*+1_) (i.e., *Y*_*k*_=0,*Y*_*k*+1_=1), matched control exposure *A*^′^ sampled from the baseline exposure levels of all subjects that are event-free at *t*_*k*_ (*Y*_*k*_=0) and have the same value for *L*_0_ as case. Sampling among these individuals is independent of baseline exposure or survival status ∙Consistency ∙Baseline conditional exchangeability ∙Positivity ∙Pr(*Y*_*k*+1_=1|*L*_0_=*l,A*_0_=1,*Y*_*k*_=0)/Pr(*Y*_*k*+1_=1|*L*_0_=*l,A*_0_=0,*Y*_*k*_=0) constant across levels *k,l*(Theorem 9, Supplementary Appendix C)	(Same as identification strategy for case-base sampling)
	Hazard ratio for per-protocol effect $\frac {\Pr (Y_{k+1}(\overline {1})=1|L_{0},...,L_{k},A_{0}=...=A_{k}=1,Y_{k}(\overline {1})=0)}{\Pr (Y_{k+1}(\overline {0})=1|L_{0},...,L_{k},A_{0}=...=A_{k}=0,Y_{k}(\overline {0})=0)}$	∙For a case with incident event in [*t*+*k,t*_*k*+1_) (i.e., *Y*_*k*_=0,*Y*_*k*+1_=1), matched control exposure *A*^′^ sampled from the baseline exposure levels *A*_0_ of all individuals who adhered to one of the protocols until *t*_*k*_ (i.e., *A*_0_=...=*A*_*k*_) and have covariate history up to *t*_*k*_. Sampling among these individuals is independent of baseline exposure or survival status ∙Consistency ∙Positivity ∙Pr(*Y*_*k*+1_=1|*L*_0_,...,*L*_*k*_,*A*_0_=...=*A*_*k*_=1,*Y*_*k*_=0)/Pr(*Y*_*k*+1_=1|*L*_0_,...,*L*_*k*_,*A*_0_=...=*A*_*k*_=0,*Y*_*k*_=0) constant across levels *k* and independent of *L*_0_,...,*L*_*k*_(Theorem 10, Supplementary Appendix C)	(Same as identification strategy for case-base sampling)

See text or Supplementary material for elaboration on assumptions

Pair matching involves assigning a single control exposure level, which we denote by *A*^′^, to every case. As for case-control studies without matching, in a case-control studies with matching an individual qualifies as a case if and only if they sustain the event of interest by the administrative study end (i.e., *Y*_*K*_=1) and adhered to one of the protocols of interest until the time of the incident event. How a matched control exposure is assigned is encoded in the sampling scheme and the assumptions of Table 2. For example, for identification of the causal marginal risk ratio under case-base sampling, *A*^′^ is sampled from all study participants whose baseline covariate value matches that of the case, independently of the participants’ baseline exposure value and whether they survive until the end of study. The matching is exact in the sense that the control exposure information is derived from an individual who has the same value for the baseline covariate as the case.

The identification strategy is the same for all results listed in Table 2. Only the case-control pairs (*A*_0_,*A*^′^) with discordant exposure values (i.e., (1,0) or (0,1)) are used. Under the stated sampling schemes and assumptions, the respective estimands are identified by the ratio of discordant pairs.

## Discussion

This paper gives a formal account of how and when causal effects can be identified in case-control studies and, as such, underpins the case-control application of Dickerman et al. [3]. Like Dickerman et al., we believe that case-control studies should generally be regarded as being nested within cohort studies. This view emphasises that the threats to the validity of cohort studies should also be considered in case-control studies. For example, in case-control applications with risk-set sampling, researchers often consider the covariate and exposure status only at, or just before, the time of the event (for cases) or the time of sampling (for controls). However, where a cohort study would require information on baseline levels or the complete treatment and covariate history of participants, one should suspect that this holds for the nested case-control study too. To gain clarity, we encourage researchers to move away from using person-years, -weeks, or -days (rather than individuals) as the default units of inference [7], and to realise that inadequately addressed deviations from a target trial may lead to bias (or departure from identifiability), regardless of whether the study that attempts to emulate it is a case-control or a cohort study [3].

What is meant by a cohort study differs between authors and contexts [8]. The term ‘cohort’ may refer to either a ‘dynamic population’, or a ‘fixed cohort’, whose “membership is defined in a permanent fashion” and “determined by a single defining event and so becomes permanent” [9]. While it may sometimes be of interest to ask what would have happened with a dynamic cohort (e.g., the residents of a country) had it been subjected to one treatment protocol versus another, the results in this paper relate to fixed cohorts.

Like the cohort studies within which they are (at least conceptually) nested, case-control studies require an explicit definition of time zero, the time at which a choice is to be made between treatment strategies or protocols of interest [3]. Given a fixed cohort, time zero is generally determined by the defining event of the cohort (e.g., first diagnosis of a particular disease or having survived one year since diagnosis). This event may occur at different calendar times for different individuals. However, while a fixed cohort may be ‘open’ to new members relative to calendar time, it is always ‘closed’ along the time axis on which all subject-specific time zeros are aligned.

In this paper, time was regarded as discrete. Since we considered arbitrary intervals between time points and because, in real-world studies, time is never measured in a truly continuous fashion, this does not represent an important limitation for practical purposes. It is however important to note that the intervals between interventions and outcome assessments (in a target trial) are an intrinsic part of the estimand that lies at the start of investigation. Careful consideration of time intervals in the design of the conceptual target trial and of the actual cohort or case-control study is therefore warranted.

We emphasize that identification and estimation are distinct steps in causal inference. Although our focus was on the former, identifying functionals often naturally translate into estimators. The task of finding the estimator with the most appealing statistical properties is not necessarily straightforward, however, and is beyond the scope of this paper.

We specifically studied two causal contrasts (i.e., pairs of interventions), one corresponding to intention-to-treat effects and the other to always-versus-never per-protocol effects of a time-varying exposure. There are of course many more causal contrasts, treatment regimes and estimands conceivable that could be of interest. We argue that also for these estimands, researchers should seek to establish identifiability before they select an estimator.

The conditions under which identifiability is to be sought for practical purposes may well include more constraints or obstacles to causal inference, such as additional missingness (e.g., outcome censoring) and measurement error, than we have considered here. While some of our results assume that hazards or hazard ratios remain constant over time, in many cases these are likely time-varying [10, 11]. There are also more case-control designs (e.g., the case-crossover design) to consider. These additional complexities and designs are beyond the scope of this paper and represent an interesting direction for future research.

The case-control family of study designs is an important yet often misunderstood tool for identifying causal relations [12–15]. Although there is much to be learned, we believe that the modern arsenal for causal inference, which includes counterfactual thinking, is well-suited to make transparent for these classical epidemiological study designs what assumptions are sufficient or necessary to endow the study results with a causal interpretation and, in turn, help resolve or prevent misunderstanding.

## Supplementary Information

**Additional file 1** Supplementary material to ‘Identification of causal effects in case-control studies’.

## Data Availability

Data sharing is not applicable to this article as no datasets were generated or analysed during the current study.
