# Supplementary material for: Identification of causal effects in case-control studies
Source: BMC Med Res Methodol. 2022 Jan 7;22:7. doi: 10.1186/s12874-021-01484-7 (PMC8742362; doi:10.1186/s12874-021-01484-7)
Supplement: Supplementary file 1 — Additional file 1 Supplementary material to ‘Identification of causal effects in case-control studies’. [file 12874_2021_1484_MOESM1_ESM.pdf]

Supplementary material to 'Identification of causal effects in case-control studies'

Table of contents

Page

Appendix A: Notation and set-up 2

Appendix B: Identification results for non-matching strategies 3

Theorem 1: Case-base sampling for marginal intention-to-treat effect *3*

Theorem 2: Case-base sampling for conditional intention-to-treat effect *5*

Theorem 3: Survivor sampling for conditional intention-to-treat effect *6*

Theorem 4: Risk-set sampling for marginal intention-to-treat effect *10*

Theorem 5: Risk-set sampling for conditional intention-to-treat effect *13*

Theorem 6: Risk-set sampling for marginal per-protocol effect *15*

Appendix C: Identification results for exact 1:M matching strategies 19

Theorem 7: Case-base sampling for marginal intention-to-treat effect *20*

Theorem 8: Survivor sampling for conditional intention-to-treat effect *21*

Theorem 9: Risk-set sampling for conditional intention-to-treat effect *22*

Theorem 10: Risk-set sampling for conditional per-protocol effect *24*

Appendix D: Parametric identification by conditional logistic regression for exact or partial 1:M matching 26

Theorem 11: Conditional logistic regression for conditional intention-to-treat effect *26*

Appendix A: Notation and set-up

We will suppose that the interest lies with the effect of a time-varying exposure that can take one of two levels at any given time on a failure time outcome. In particular, we consider a strictly increasing sequence $(t_0, t_1, ..., t_K)$ of $K+1$ time points (with $t_{K+1} = -t_{-1} = +\infty$ for notational convenience). For $k = 0, 1, ..., K-1$, let A_k denote the level of time-varying exposure of interest at t_k. We denote the history of any stochastic sequence $(X_0, X_1, ..., X_{K-1})$ up to and including t_k by $\overline{X}_k = (X_0, X_1 ..., X_k)$ for $k = 0, 1, ..., K-1$ (and let $\overline{X} = \overline{X}_{K-1}$ and $\overline{X}_{-1} = 0$ for notational convenience). For example, $\overline{A} = (A_0, A_1, ..., A_{K-1})$. Denote by $T(\overline{a})$ the counterfactual time elapsed until the event of interest since t_0 that would have been realised had $\overline{A}$ been set to $\overline{a}$, and let $Y_k(\overline{a}) = I(T(\overline{a}) < t_k)$ for $k = 0, 1, ..., K$, where I represents the indicator function. By convention, we stipulate that for all k, $Y_k(\overline{a})$ is invariant to the kth through $K-1$th elements of $\overline{a}$ (i.e., current survival status is not affected by future exposures). With slight abuse of notation, for $k = 0, 1..., K$, we let $Y_k(a_0)$ denote the outcome that would have been realised had (only) A_0 been set to a_0.

Consistency

For theorems about per-protocol effects, we assume consistency of the form: for $k = 1, ..., K$ and all $\overline{a}$, $Y_k(\overline{a}) = Y_k$ if $a_l = A_l$ for all $l = 0, ..., k-1$ such that $Y_l = 0$. For theorems about intention-to-treat effects, a weaker condition is sufficient and assumed: for $k = 1, ..., K$ and $a = 0, 1$, $Y_k(a) = Y_k$ if $a = A_0$. The assumption may be further relaxed for theorems in which the estimand does not involve $Y_k(a)$, $k < K$: for $a = 0, 1$, $Y_K(a) = Y_K$ if $a = A_0$.

Conditional exchangeability

We also consider a sequence of variables $\overline{L} = (L_0, L_1, ..., L_{K-1})$ that satisfies one of the following conditions:

$$\forall k, \forall \overline{a} : (Y_{k+1}(\overline{a}), ..., Y_K(\overline{a})) \perp\!\!\!\perp A_k | Y_k(\overline{a}) = 0, \overline{L}_k, \overline{A}_{k-1} = \overline{a}_{k-1}, \quad \text{(sequential conditional exchangeability, SCE)}$$

where $\overline{a}_{k-1}$ is understood to represent the $(k-1)$th through $(K-1)$th elements of $\overline{a}$, or

$$\forall a_0 : (Y_1(a_0), ..., Y_K(a_0)) \perp\!\!\!\perp A_0 | L_0, \quad \text{(baseline conditional exchangeability, BCE)}$$

although sometimes a weaker form of BCE suffices: $\forall a_0 : Y_K(a_0) \perp\!\!\!\perp A_0 | L_0$.

Positivity

For the theorems that follow, we assume positivity to preclude division by zero and undefined conditional probabilities, so that the weights that we will encounter are finite and strictly greater than 1. The assumption can sometimes be relaxed if we are willing to interpolate or extrapolate under (parametric) modelling assumptions.

Appendix B: Identification results for non-matching strategies

Intention-to-treat effect

For simplicity, it is assumed below that the covariates are discrete. The results can however be extended to more general distributions.

Theorem 1 (Case-base sampling for marginal intention-to-treat effect) *Suppose BCE holds as well as*

$$\Pr(S=1|L_0,A_0)=\Pr(S=1)=\delta \tag{S1}$$

for some $\delta \in (0,1]$. *Then,*

$$\frac{\dfrac{\mathbb{E}\big[I(A_0=1)W|Y_K=1\big]}{\mathbb{E}\big[I(A_0=0)W|Y_K=1\big]}}{\dfrac{\mathbb{E}\big[I(A_0=1)W|S=1\big]}{\mathbb{E}\big[I(A_0=0)W|S=1\big]}}=\frac{\Pr(Y_K(1)=1)}{\Pr(Y_K(0)=1)},$$

where

$$W=\left.\frac{1}{\Pr(A_0=a|L_0,S=1)}\right|_{a=A_0},$$

Proof First, observe that $\Pr(A_0=a|L_0,S=1)=\Pr(A_0=a|L_0)$ for $a=0,1$, because

$$\begin{aligned}\Pr(A_0=a|L_0,S=1)&=\frac{\Pr(S=1|L_0,A_0=a)\Pr(A_0=a|L_0)}{\Pr(S=1|L_0)}\\&=\frac{\delta}{\delta}\Pr(A_0=a|L_0) && \text{(by S1)}\\&=\Pr(A_0=a|L_0)\end{aligned}$$

Hence,

$$W=\left.\frac{1}{\Pr(A_0=a|L_0)}\right|_{a=A_0}.$$

Now, consider the numerator of the left-hand side of the main equation in Theorem 1 and note that, because of the above, we have

$$\begin{aligned}\frac{\mathbb{E}\big[I(A_0=1)W|Y_K=1\big]}{\mathbb{E}\big[I(A_0=0)W|Y_K=1\big]}&=\frac{\sum_{y=0}^{1}\mathbb{E}\big[I(A_0=1)WY_K|Y_K=y\big]\Pr(Y_K=y)}{\sum_{y=0}^{1}\mathbb{E}\big[I(A_0=0)WY_K|Y_K=y\big]\Pr(Y_K=y)}\\&=\frac{\mathbb{E}\big[I(A_0=1)WY_K\big]}{\mathbb{E}\big[I(A_0=0)WY_K\big]}\\&=\frac{\mathbb{E}\big[WY_K|A_0=1\big]\Pr(A_0=1)}{\mathbb{E}\big[WY_K|A_0=0\big]\Pr(A_0=0)},\end{aligned}$$

where

$$\begin{aligned}
\mathbb{E}\big[WY_K|A_0=a\big] &= \mathbb{E}\big\{\mathbb{E}\big[WY_K|L_0,A_0=a\big]|A_0=a\big\} \\
&= \sum_l \frac{\Pr(Y_K=1|L_0=l,A_0=a)\Pr(L_0=l|A_0=a)}{\Pr(A_0=a|L_0=l)} \\
&= \sum_l \frac{\Pr(Y_K(a)=1|L_0=l,A_0=a)\Pr(L_0=l|A_0=a)}{\Pr(A_0=a|L_0=l)} \\
&\qquad\qquad\qquad\qquad\qquad\qquad\qquad\qquad\qquad \text{(by consistency)} \\
&= \sum_l \frac{\Pr(Y_K(a)=1|L_0=l)\Pr(L_0=l|A_0=a)}{\Pr(A_0=a|L_0=l)} \\
&\qquad\qquad\qquad\qquad\qquad \text{(by baseline conditional exchangeability)} \\
&= \sum_l \frac{\Pr(Y_K(a)=1|L_0=l)\Pr(A_0=a|L_0=l)\Pr(L_0=l)}{\Pr(A_0=a|L_0=l)\Pr(A_0=a)} \\
&= \frac{1}{\Pr(A_0=a)}\sum_l \Pr(Y_K(a)=1,L_0=l) \\
&= \frac{\Pr(Y_K(a)=1)}{\Pr(A_0=a)},
\end{aligned}$$

so that

$$\frac{\mathbb{E}\big[I(A_0=1)W|Y_K=1\big]}{\mathbb{E}\big[I(A_0=0)W|Y_K=1\big]} = \frac{\Pr(Y_K(1)=1)}{\Pr(Y_K(0)=1)}.$$

Next, consider the denominator of the left-hand side of the main equation in Theorem 1 and observe that

$$\frac{\mathbb{E}\big[I(A_0=1)W|S=1\big]}{\mathbb{E}\big[I(A_0=0)W|S=1\big]} = \frac{\mathbb{E}\big[I(A_0=1)WS\big]}{\mathbb{E}\big[I(A_0=0)WS\big]} = \frac{\mathbb{E}\big[WS|A_0=1\big]\Pr(A_0=1)}{\mathbb{E}\big[WS|A_0=0\big]\Pr(A_0=0)},$$

where

$$\begin{aligned}
\mathbb{E}\big[WS|A_0=a\big] &= \mathbb{E}\{\mathbb{E}\big[WS|L_0,A_0=a\big]|A_0=a\} \\
&= \sum_l \frac{\Pr(S=1|L_0,A_0=a)\Pr(L_0=l|A_0=a)}{\Pr(A_0=a|L_0=l)} \\
&= \sum_l \frac{\delta\Pr(L_0=l|A_0=a)}{\Pr(A_0=a|L_0=l)} \qquad\qquad\qquad\qquad \text{(by S1)} \\
&= \frac{\delta}{\Pr(A_0=a)}\sum_l \Pr(L_0=l) \\
&= \frac{\delta}{\Pr(A_0=a)},
\end{aligned}$$

so that

$$\frac{\mathbb{E}\big[I(A_0=1)W|S=1\big]}{\mathbb{E}\big[I(A_0=0)W|S=1\big]} = 1.$$

It follows that

$$\frac{\frac{\mathbb{E}\big[I(A_0=1)W|Y_K=1\big]}{\mathbb{E}\big[I(A_0=0)W|Y_K=1\big]}}{\frac{\mathbb{E}\big[I(A_0=1)W|S=1\big]}{\mathbb{E}\big[I(A_0=0)W|S=1\big]}} = \frac{\Pr(Y_K(1)=1)}{\Pr(Y_K(0)=1)}.$$

□

Theorem 2 (Case-base sampling for conditional intention-to-treat effect) *Suppose BCE hold as well as S1, or the weaker version* $\Pr(S=1|L_0,A_0)=\Pr(S=1|L_0)=\delta_{L_0}\in(0,1]$. *Then,*

$$\frac{\frac{\mathbb{E}\big[I(A_0=1)|L_0,Y_K=1\big]}{\mathbb{E}\big[I(A_0=0)|L_0,Y_K=1\big]}}{\frac{\mathbb{E}\big[I(A_0=1)|L_0,S=1\big]}{\mathbb{E}\big[I(A_0=0)|L_0,S=1\big]}} = \frac{\Pr(Y_K(1)=1|L_0)}{\Pr(Y_K(0)=1|L_0)}.$$

Proof We have

$$\begin{aligned}\frac{\mathbb{E}\big[I(A_0=1)|L_0,Y_K=1\big]}{\mathbb{E}\big[I(A_0=0)|L_0,Y_K=1\big]} &= \frac{\sum_{y=0}^{1}\mathbb{E}\big[I(A_0=1)Y_K|L_0,Y_K=y\big]\Pr(Y_K=y|L_0)}{\sum_{y=0}^{1}\mathbb{E}\big[I(A_0=0)Y_K|L_0,Y_K=y\big]\Pr(Y_K=y|L_0)}\\ &= \frac{\mathbb{E}\big[I(A_0=1)Y_K|L_0\big]}{\mathbb{E}\big[I(A_0=0)Y_K|L_0\big]}\\ &= \frac{\mathbb{E}\big[Y_K|L_0,A_0=1\big]\Pr(A_0=1|L_0)}{\mathbb{E}\big[Y_K|L_0,A_0=0\big]\Pr(A_0=0|L_0)}\\ &= \frac{\mathbb{E}\big[Y_K(1)|L_0,A_0=1\big]\Pr(A_0=1|L_0)}{\mathbb{E}\big[Y_K(0)|L_0,A_0=0\big]\Pr(A_0=0|L_0)}\\ &\qquad\qquad\text{(by consistency)}\\ &= \frac{\mathbb{E}\big[Y_K(1)|L_0\big]\Pr(A_0=1|L_0)}{\mathbb{E}\big[Y_K(0)|L_0\big]\Pr(A_0=0|L_0)}.\\ &\qquad\qquad\text{(by baseline conditional exchangeability)}\end{aligned}$$

Also,

$$\begin{aligned}\frac{\mathbb{E}\big[I(A_0=1)|L_0,S=1\big]}{\mathbb{E}\big[I(A_0=0)|L_0,S=1\big]} &= \frac{\mathbb{E}\big[I(A_0=1)S|L_0\big]}{\mathbb{E}\big[I(A_0=0)S|L_0\big]}\\ &= \frac{\mathbb{E}\big[S|L_0,A_0=1\big]\Pr(A_0=1|L_0)}{\mathbb{E}\big[S|L_0,A_0=0\big]\Pr(A_0=0|L_0)}\\ &= \frac{\delta_{L_0}\Pr(A_0=1|L_0)}{\delta_{L_0}\Pr(A_0=0|L_0)}\\ &\text{(under the assumption that }\Pr(S=1|L_0,A_0)=\Pr(S=1|L_0)=\delta_{L_0}\in(0,1])\\ &= \frac{\Pr(A_0=1|L_0)}{\Pr(A_0=0|L_0)}.\end{aligned}$$

It immediately follows that

$$\frac{\frac{\mathbb{E}\big[I(A_0=1)|L_0,Y_K=1\big]}{\mathbb{E}\big[I(A_0=0)|L_0,Y_K=1\big]}}{\frac{\mathbb{E}\big[I(A_0=1)|L_0,S=1\big]}{\mathbb{E}\big[I(A_0=0)|L_0,S=1\big]}} = \frac{\Pr(Y_K(1)=1|L_0)}{\Pr(Y_K(0)=1|L_0)}.$$

□

Corollary 1 *If in addition to the conditions of Theorem 2,*

$$\frac{\Pr(Y_K=1|L_0=l,A_0=1)}{\Pr(Y_K=1|L_0=l,A_0=0)} = \theta \qquad \text{(homogeneity condition H1)}$$

for all l and some constant θ, then

$$\frac{\frac{\mathbb{E}\big[I(A_0=1)|L_0,Y_K=1\big]}{\mathbb{E}\big[I(A_0=0)|L_0,Y_K=1\big]}}{\frac{\mathbb{E}\big[I(A_0=1)|L_0,S=1\big]}{\mathbb{E}\big[I(A_0=0)|L_0,S=1\big]}} = \frac{\Pr(Y_K(1)=1)}{\Pr(Y_K(0)=1)},$$

because of the collapsibility of the risk ratio.

Theorem 3 (Survivor sampling for conditional intention-to-treat effect) *Suppose BCE holds as well as*

$$\Pr(S=1|L_0,A_0,Y_K) = \Pr(S=1|L_0,Y_K) = \delta_{L_0}\times(1-Y_K) \qquad \text{(S2)}$$

for some $\delta_{L_0}\in(0,1]$. Then,

$$\frac{\frac{\mathbb{E}\big[I(A_0=1)|L_0,Y_K=1\big]}{\mathbb{E}\big[I(A_0=0)|L_0,Y_K=1\big]}}{\frac{\mathbb{E}\big[I(A_0=1)|L_0,S=1\big]}{\mathbb{E}\big[I(A_0=0)|L_0,S=1\big]}} = \frac{\text{Odds}(Y_K(1)=1|L_0)}{\text{Odds}(Y_K(0)=1|L_0)}.$$

Proof First, consider the numerator of the left-hand side of the equation in Theorem 3 and observe

$$\begin{aligned}\frac{\mathbb{E}\big[I(A_0=1)|L_0,Y_K=1\big]}{\mathbb{E}\big[I(A_0=0)|L_0,Y_K=1\big]} &= \frac{\Pr(Y_K=1|L_0,A_0=1)}{\Pr(Y_K=1|L_0,A_0=0)}\text{Odds}(A_0=1|L_0)\\ &= \frac{\Pr(Y_K(1)=1|L_0,A_0=1)}{\Pr(Y_K(1)=1|L_0,A_0=0)}\text{Odds}(A_0=1|L_0)\\ &\qquad\text{(by consistency)}\\ &= \frac{\Pr(Y_K(1)=1|L_0)}{\Pr(Y_K(1)=1|L_0)}\text{Odds}(A_0=1|L_0).\\ &\qquad\text{(by baseline conditional exchangeability)}\end{aligned}$$

Next, consider the denominator and observe that

$$\begin{aligned}
\frac{\mathbb{E}\big[I(A_0=1)|L_0,S=1\big]}{\mathbb{E}\big[I(A_0=0)|L_0,S=1\big]} &= \frac{\mathbb{E}\big[I(A_0=1)S|L_0\big]}{\mathbb{E}\big[I(A_0=0)S|L_0\big]} \\
&= \frac{\mathbb{E}\big[S|L_0,A_0=1\big]}{\mathbb{E}\big[S|L_0,A_0=0\big]}\text{Odds}(A_0=1|L_0) \\
&= \frac{\delta_{L_0}\Pr(Y_K=0|L_0,A_0=1)}{\delta_{L_0}\Pr(Y_K=0|L_0,A_0=0)}\text{Odds}(A_0=1|L_0) \\
&\qquad\qquad\qquad\qquad\qquad\qquad\text{(by S2)} \\
&= \frac{\Pr(Y_K(1)=0|L_0,A_0=1)}{\Pr(Y_K(0)=0|L_0,A_0=0)}\text{Odds}(A_0=1|L_0) \\
&\qquad\qquad\qquad\qquad\qquad\qquad\text{(by consistency)} \\
&= \frac{\Pr(Y_K(1)=0|L_0)}{\Pr(Y_K(0)=0|L_0)}\text{Odds}(A_0=1|L_0). \\
&\qquad\qquad\text{(by baseline conditional exchangeability)}
\end{aligned}$$

It follows that

$$\frac{\dfrac{\mathbb{E}\big[I(A_0=1)|L_0,Y_K=1\big]}{\mathbb{E}\big[I(A_0=0)|L_0,Y_K=1\big]}}{\dfrac{\mathbb{E}\big[I(A_0=1)|L_0,S=1\big]}{\mathbb{E}\big[I(A_0=0)|L_0,S=1\big]}} = \frac{\text{Odds}(Y_K(1)=1|L_0)}{\text{Odds}(Y_K(0)=1|L_0)}.$$

□

Remark (Remark to Theorem 3) *Under BCE, the stronger version of S2,*

$$\Pr(S=1|L_0,A_0,Y_K) = \Pr(S=1|Y_K) = \delta\times(1-Y_K) \qquad \text{(S2}^*\text{)}$$

for some $\delta\in(0,1]$ *and with*

$$W = \left.\frac{1}{\Pr(A_0=a|L_0)}\right|_{a=A_0},$$

we have

$$\frac{\dfrac{\mathbb{E}\big[I(A_0=1)W|Y_K=1\big]}{\mathbb{E}\big[I(A_0=0)W|Y_K=1\big]}}{\dfrac{\mathbb{E}\big[I(A_0=1)W|S=1\big]}{\mathbb{E}\big[I(A_0=0)W|S=1\big]}} = \frac{\text{Odds}(Y_K(1)=1)}{\text{Odds}(Y_K(0)=1)} \qquad (1)$$

(see proof below). However, from

$$\begin{aligned}
\Pr(A_0=a|L_0,S=1) &= \frac{\Pr(S=1|L_0,A_0=a)\Pr(A_0=a|L_0)}{\Pr(S=1|L_0)} \\
&= \frac{\delta\Pr(Y_K=0|L_0,A_0=a)\Pr(A_0=a|L_0)}{\delta\Pr(Y_K=0|L_0)} \qquad \text{(by S2}^*\text{)}
\end{aligned}$$

$$= \Pr(A_0 = a | L_0, Y_K = 0),$$

it follows that the weights W above are not identified by

$$\left. \frac{1}{\Pr(A_0 = a | L_0, S = 1)} \right|_{a = A_0}$$

when $Y_K \not\perp\!\!\!\perp A_0 | L_0$. (However, $\Pr(A_0 = a | L_0, S = 1)$ approximates $\Pr(A_0 = a | L_0)$ under a rare event assumption.) In fact, the target marginal odds ratio is not identifiable, under BCE and S2 with unknown δ, from the available data distribution, which is formed by the distribution of $(L_0, A_0, Y_K, S) | (Y_K = 1 \vee S = 1)$. A proof is given below.*

Proof of (1) *under stated conditions* As shown in the proof to Theorem 1,

$$\frac{\mathbb{E}\big[I(A_0 = 1) W | Y_K = 1\big]}{\mathbb{E}\big[I(A_0 = 0) W | Y_K = 1\big]} = \frac{\Pr(Y_K(1) = 1)}{\Pr(Y_K(0) = 1)}.$$

Now,

$$\frac{\mathbb{E}\big[I(A_0 = 1) W | S = 1\big]}{\mathbb{E}\big[I(A_0 = 0) W | S = 1\big]} = \frac{\mathbb{E}\big[I(A_0 = 1) WS\big]}{\mathbb{E}\big[I(A_0 = 0) WS\big]} = \frac{\mathbb{E}\big[WS | A_0 = 1\big] \Pr(A_0 = 1)}{\mathbb{E}\big[WS | A_0 = 0\big] \Pr(A_0 = 0)},$$

where

$$\begin{aligned}
\mathbb{E}\big[WS | A_0 = a\big] &= \mathbb{E}\{\mathbb{E}\big[WS | L_0, A_0 = a\big] | A_0 = a\} \\
&= \sum_l \frac{\Pr(S = 1 | L_0, A_0 = a) \Pr(L_0 = l | A_0 = a)}{\Pr(A_0 = a | L_0 = l)} \\
&= \sum_l \frac{\delta \Pr(Y_K = 0 | L_0 = l, A_0 = a) \Pr(L_0 = l | A_0 = a)}{\Pr(A_0 = a | L_0 = l)} \\
&\hspace{20em} \text{(by S2}^*\text{)} \\
&= \frac{\delta}{\Pr(A_0 = a)} \sum_l \Pr(Y_K = 0 | L_0 = l, A_0 = a) \Pr(L_0 = l) \\
&= \frac{\delta}{\Pr(A_0 = a)} \sum_l \Pr(Y_K(a) = 0 | L_0 = l, A_0 = a) \Pr(L_0 = l) \\
&\hspace{20em} \text{(by consistency)} \\
&= \frac{\delta}{\Pr(A_0 = a)} \sum_l \Pr(Y_K(a) = 0, L_0 = l) \\
&\hspace{12em} \text{(by baseline conditional exchangeability)} \\
&= \frac{\delta \Pr(Y_K(a) = 0)}{\Pr(A_0 = a)},
\end{aligned}$$

so that

$$\frac{\mathbb{E}\big[I(A_0 = 1) W | S = 1\big]}{\mathbb{E}\big[I(A_0 = 0) W | S = 1\big]} = \frac{\Pr(Y_K(1) = 0)}{\Pr(Y_K(0) = 0)}$$

and, in turn,

$$\frac{\dfrac{\mathbb{E}\big[I(A_0 = 1)W|Y_K = 1\big]}{\mathbb{E}\big[I(A_0 = 0)W|Y_K = 1\big]}}{\dfrac{\mathbb{E}\big[I(A_0 = 1)W|S = 1\big]}{\mathbb{E}\big[I(A_0 = 0)W|S = 1\big]}} = \frac{\text{Odds}(Y_K(1) = 1)}{\text{Odds}(Y_K(0) = 1)}.$$

□

Proof of nonidentifiability of target marginal odds ratio under stated conditions Consider two distributions of (L_0, A_0, Y_K, S) satisfying S2*, each characterised by the following conditionals:

$$\begin{aligned} Y_K &\sim \text{Bernoulli}(\alpha), \\ S|Y_K &\sim \text{Bernoulli}(\delta \times (1 - Y_K)), \\ L_0|Y_K, S \sim L_0|Y_K &\sim \text{Bernoulli}(5/10 - 2/10 \times Y_K), \\ A_0|L_0, Y_K, S \sim A_0|L_0, Y_K &\sim \text{Bernoulli}(3/10 + 2/10 \times L_0 + 3/10 \times Y_K). \end{aligned}$$

The parameter values of the distributions are given in the table below.

Parameter	Distribution 1	Distribution 2
α	1/10	2/10
δ	1/10	9/40

Now, for all $l, a, y, s \in \{0, 1\}$,

$$\begin{aligned} &\Pr(L_0 = l, A_0 = a, Y_K = y, S = s|Y_K = 1 \vee S = 1) \\ &= \frac{\Pr(L_0 = l, A_0 = a, Y_K = y, S = s, Y_K = 1 \vee S = 1)}{\Pr(Y_K = 1 \wedge S = 0) + \Pr(Y_K = 0 \wedge S = 1) + \Pr(Y_K = 1 \wedge S = 1)} \\ &= \frac{I(y = 1 \vee s = 1) \Pr(L_0 = l, A_0 = a, Y_K = y, S = s)}{\Pr(Y_K = 1) + \delta \Pr(Y_K = 0)} \\ &= I(y = 1 \vee s = 1) \frac{\Pr(L_0 = l, A_0 = a|Y_K = y) \Pr(S = s|Y_K = y) \Pr(Y_K = y)}{\alpha + \delta(1 - \alpha)} \\ &= \begin{cases} \Pr(L_0 = l, A_0 = a|Y_K = 0)\left(1 - \dfrac{\alpha}{\alpha + \delta(1 - \alpha)}\right) & \text{if } y = 0 \wedge s = 1, \\ \Pr(L_0 = l, A_0 = a|Y_K = 1)\dfrac{\alpha}{\alpha + \delta(1 - \alpha)} & \text{if } y = 1 \wedge s = 0, \\ 0 & \text{otherwise,} \end{cases} \end{aligned}$$

where

$$\frac{\alpha}{\alpha + \delta(1 - \alpha)} = 10/19$$

under Distribution 1 and under Distribution 2. Hence, Distribution 1 and 2 imply the same available data distribution.

However, as we now show, the distributions imply different target marginal odds ratios. Since

$$
\begin{aligned}
\Pr(Y_K(a)=1) &= \sum_{l=0}^{1} \Pr(Y_K(a)=1|L_0=l)\Pr(L_0=l) \\
&= \sum_{l=0}^{1} \Pr(Y_K(a)=1|L_0=l, A_0=a)\Pr(L_0=l) \qquad \text{(by BCE)} \\
&= \sum_{l=0}^{1} \Pr(Y_K=1|L_0=l, A_0=a)\Pr(L_0=l) \quad \text{(by consistency)} \\
&= \sum_{l=0}^{1} \frac{\Pr(L_0=l, A_0=a|Y_K=1)\Pr(Y_K=1)}{\Pr(L_0=l, A_0=a)} \sum_{y=0}^{1} \Pr(L_0=l|Y_K=y)\Pr(Y_K=y) \\
&= \sum_{l=0}^{1} \left(1+\frac{\Pr(L_0=l, A_0=a|Y_K=0)\Pr(Y_K=0)}{\Pr(L_0=l, A_0=a|Y_K=1)\Pr(Y_K=1)}\right)^{-1} \sum_{y=0}^{1} \Pr(L_0=l|Y_K=y)\Pr(Y_K=y)
\end{aligned}
$$

for $a=0,1$, we have

$$
\begin{aligned}
\Pr(Y_K(1)=1) &= \frac{5+2\alpha}{10+(25/7)/\text{odds}(\alpha)} + \frac{5-2\alpha}{10+(125/12)/\text{odds}(\alpha)} \quad \text{and} \\
\Pr(Y_K(0)=1) &= \frac{5+2\alpha}{10+(25/2)/\text{odds}(\alpha)} + \frac{5-2\alpha}{10+(125/3)/\text{odds}(\alpha)},
\end{aligned}
$$

so that

$$
\frac{\text{Odds}(Y_K(1)=1)}{\text{Odds}(Y_K(0)=1)} = \begin{cases} \frac{587,791}{167,166} \approx 3.5 & \text{under Distribution 1,} \\ \frac{512,539}{148,789} \approx 3.4 & \text{under Distribution 2.} \end{cases}
$$

Hence, we found an available data distribution that is compatible with more than one value of the target marginal odds ratio. This concludes the proof. □

Theorem 4 (Risk-set sampling for marginal intention-to-treat effect) *Suppose BCE holds as well as*

$$
\Pr(S_k=1|L_0, A_0, Y_k) = \Pr(S_k=1|Y_k) = \delta \times (1-Y_k), \tag{S3}
$$

for some $\delta \in (0,1]$*. If*

$$
\Pr(Y_{k+1}(a)=1|Y_k(a)=0) = \theta_a \tag{H2}
$$

for $a=0,1$ *and some constants* θ_0, θ_1*, then*

$$
\frac{\dfrac{\mathbb{E}\big[I(A_0=1)W|Y_K=1\big]}{\mathbb{E}\big[I(A_0=0)W|Y_K=1\big]}}{\dfrac{\mathbb{E}\big[I(A_0=1)W\sum_{k=0}^{K-1}S_k\big]}{\mathbb{E}\big[I(A_0=0)W\sum_{k=0}^{K-1}S_k\big]}} = \frac{\Pr(Y_{k+1}(1)=1|Y_{k+1}(1)=0)}{\Pr(Y_{k+1}(0)=1|Y_{k+1}(0)=0)},
$$

where

$$W = \left.\frac{1}{\Pr(A_0 = a|L_0, S_0 = 1)}\right|_{a=A_0},$$

Proof First, observe that $\Pr(A_0 = a|L_0, S_0 = 1) = \Pr(A_0 = a|L_0)$ for $a = 0, 1$, because

$$\begin{aligned}
\Pr(A_0 = a|L_0, S_0 = 1) &= \frac{\Pr(S_0 = 1|L_0, A_0 = a)\Pr(A_0 = a|L_0)}{\Pr(S_0 = 1|L_0)} \\
&= \frac{\delta}{\delta}\Pr(A_0 = a|L_0) && \text{(by S3)} \\
&= \Pr(A_0 = a|L_0)
\end{aligned}$$

Hence,

$$W = \left.\frac{1}{\Pr(A_0 = a|L_0)}\right|_{a=A_0}.$$

For the numerator of the main result of Theorem 4, we thus have

$$\begin{aligned}
\frac{\mathbb{E}\big[I(A_0 = 1)W|Y_K = 1\big]}{\mathbb{E}\big[I(A_0 = 0)W|Y_K = 1\big]} &= \frac{\mathbb{E}\big[I(A_0 = 1)WY_K\big]}{\mathbb{E}\big[I(A_0 = 0)WY_K\big]} \\
&= \frac{\mathbb{E}\big[WY_K|A_0 = 1\big]\Pr(A_0 = 1)}{\mathbb{E}\big[WY_K|A_0 = 0\big]\Pr(A_0 = 0)},
\end{aligned}$$

where

$$\begin{aligned}
\mathbb{E}\big[WY_K|A_0 = a\big] &= \mathbb{E}\big\{\mathbb{E}\big[WY_K|L_0, A_0 = a\big]|A_0 = a\big\} \\
&= \sum_l \frac{\Pr(Y_K = 1|L_0 = l, A_0 = a)\Pr(L_0 = l|A_0 = a)}{\Pr(A_0 = a|L_0 = l)} \\
&= \sum_l \frac{\Pr(Y_K(a) = 1|L_0 = l, A_0 = a)\Pr(L_0 = l|A_0 = a)}{\Pr(A_0 = a|L_0 = l)} \\
& && \text{(by consistency)} \\
&= \sum_l \frac{\Pr(Y_K(a) = 1|L_0 = l)\Pr(L_0 = l|A_0 = a)}{\Pr(A_0 = a|L_0 = l)} \\
& && \text{(by baseline conditional exchangeability)} \\
&= \sum_l \frac{\Pr(Y_K(a) = 1|L_0 = l)\Pr(A_0 = a|L_0 = l)\Pr(L_0 = l)}{\Pr(A_0 = a|L_0 = l)\Pr(A_0 = a)} \\
&= \frac{1}{\Pr(A_0 = a)}\sum_l \Pr(Y_K(a) = 1, L_0 = l) \\
&= \frac{\Pr(Y_K(a) = 1)}{\Pr(A_0 = a)},
\end{aligned}$$

so that

$$\frac{\mathbb{E}\big[I(A_0 = 1)W|Y_K = 1\big]}{\mathbb{E}\big[I(A_0 = 0)W|Y_K = 1\big]} = \frac{\Pr(Y_K(1) = 1)}{\Pr(Y_K(0) = 1)}$$

$$
\begin{aligned}
&= \frac{\sum_{k=0}^{K-1} \Pr(Y_{k+1}(1)=1, Y_k(1)=0)}{\sum_{k=0}^{K-1} \Pr(Y_{k+1}(0)=1, Y_k(0)=0)} \\
&= \frac{\sum_{k=0}^{K-1} \Pr(Y_{k+1}(1)=1|Y_k(1)=0)\Pr(Y_k(1)=0)}{\sum_{k=0}^{K-1} \Pr(Y_{k+1}(0)=1|Y_k(0)=0)\Pr(Y_k(0)=0)} \\
&= \frac{\sum_{k=0}^{K-1} \theta_1 \Pr(Y_k(1)=0)}{\sum_{k=0}^{K-1} \theta_0 \Pr(Y_k(0)=0)} \qquad \text{(by H2)} \\
&= \frac{\theta_1}{\theta_0}\frac{\sum_{k=0}^{K-1} \Pr(Y_k(1)=0)}{\sum_{k=0}^{K-1} \Pr(Y_k(0)=0)}
\end{aligned}
$$

For the denominator, we have

$$
\frac{\mathbb{E}\big[I(A_0=1)W\sum_{k=0}^{K-1} S_k\big]}{\mathbb{E}\big[I(A_0=0)W\sum_{k=0}^{K-1} S_k\big]} = \frac{\mathbb{E}\big[W\sum_{k=0}^{K-1} S_k|A_0=1\big]\Pr(A_0=1)}{\mathbb{E}\big[W\sum_{k=0}^{K-1} S_k|A_0=0\big]\Pr(A_0=0)},
$$

where

$$
\begin{aligned}
\mathbb{E}\big[W\textstyle\sum_{k=0}^{K-1} S_k|A_0=a\big] &= \textstyle\sum_{k=0}^{K-1}\mathbb{E}\big\{\mathbb{E}\big[WS_k|L_0,A_0=a\big]|A_0=a\big\} \\
&= \sum_{k=0}^{K-1}\sum_l \frac{\Pr(S_k=1|L_0,A_0=a)\Pr(L_0=l|A_0=a)}{\Pr(A_0=a|L_0=l)} \\
&= \sum_{k=0}^{K-1}\sum_l \frac{\delta\Pr(Y_k=0|L_0=l,A_0=a)\Pr(L_0=l|A_0=a)}{\Pr(A_0=a|L_0=l)} \\
&\qquad \text{(by S3)} \\
&= \sum_{k=0}^{K-1}\sum_l \frac{\delta\Pr(Y_k=0|L_0=l,A_0=a)\Pr(L_0=l)}{\Pr(A_0=a)} \\
&= \sum_{k=0}^{K-1}\sum_l \frac{\delta\Pr(Y_k(a)=0|L_0=l,A_0=a)\Pr(L_0=l)}{\Pr(A_0=a)} \\
&\qquad \text{(by consistency)} \\
&= \sum_{k=0}^{K-1}\sum_l \frac{\delta\Pr(Y_k(a)=0|L_0=l)\Pr(L_0=l)}{\Pr(A_0=a)} \\
&\qquad \text{(by baseline conditional exchangeability)} \\
&= \frac{1}{\Pr(A_0=a)}\sum_{k=0}^{K-1}\sum_l \delta\Pr(Y_k(a)=0,L_0=l) \\
&= \frac{1}{\Pr(A_0=a)}\sum_{k=0}^{K-1} \delta\Pr(Y_k(a)=0),
\end{aligned}
$$

so that

$$
\begin{aligned}
\frac{\mathbb{E}\big[I(A_0=1)W\sum_{k=0}^{K-1} S_k\big]}{\mathbb{E}\big[I(A_0=0)W\sum_{k=0}^{K-1} S_k\big]} &= \frac{\sum_{k=0}^{K-1}\delta\Pr(Y_k(1)=0)}{\sum_{k=0}^{K-1}\delta\Pr(Y_k(0)=0)} \\
&= \frac{\sum_{k=0}^{K-1}\Pr(Y_k(1)=0)}{\sum_{k=0}^{K-1}\Pr(Y_k(0)=0)}.
\end{aligned}
$$

It follows that

$$\frac{\dfrac{\mathbb{E}\big[I(A_0=1)W|Y_K=1\big]}{\mathbb{E}\big[I(A_0=0)W|Y_K=1\big]}}{\dfrac{\mathbb{E}\big[I(A_0=1)W\sum_{k=0}^{K-1}S_k\big]}{\mathbb{E}\big[I(A_0=0)W\sum_{k=0}^{K-1}S_k\big]}}=\frac{\Pr(Y_{k+1}(1)=1|Y_k(1)=0)}{\Pr(Y_{k+1}(0)=1|Y_k(0)=0)}.$$

□

Remark (Remark to Theorem 4) *Condition S3 holds if, for some constant* δ_k^*,

$$\left.\begin{aligned}\Pr(S_k=1)=\delta_k^*\Pr(Y_{k+1}=1,Y_k=0),\\ S_k \perp\!\!\!\perp (L_0,A_0,\overline{Y}_k)|Y_k=0,\\ \Pr(S_k=1|Y_k=1)=0.\end{aligned}\right\}\tag{S3*}$$

The first requirement of S3 essentially means that the frequency of incident cases in the kth window is proportional to the frequency of controls selected in this window. Under S3*, S3 is met with* $\delta=\delta_k^*\Pr(Y_{k+1}=1|Y_k=0)$, *because*

$$\begin{aligned}\Pr(S_k=1|L_0,A_0,\overline{Y}_k)&=\Pr(S_k=1|Y_k)\\&=\Pr(S_k=1|Y_k=0)\times(1-Y_k)\\&=\Pr(S_k=1|Y_k=0)\times(1-Y_k)\\&=\frac{\Pr(S_k=1)}{\Pr(Y_k=0)}\times(1-Y_k)\\&=\frac{\delta_k^*\Pr(Y_{k+1}=1,Y_k=0)}{\Pr(Y_k=0)}\times(1-Y_k)\\&=\delta_k^*\Pr(Y_{k+1}=1|Y_k=0)\times(1-Y_k).\end{aligned}$$

Therefore, stipulating that δ_k^* *is k-invariant is to state that* $\Pr(Y_{k+1}=1|Y_k=0)$ *is constant for* $k=0,...,K-1$.

Theorem 5 (Risk-set sampling for conditional intention-to-treat effect) *Suppose BCE holds as well as S3, or the weaker version* $\Pr(S_k=1|L_0,A_0,Y_k)=\Pr(S_k=1|L_0,Y_k)=\delta_{L_0}\times(1-Y_k),\ \delta_{L_0}\in(0,1]$. *If*

$$\Pr(Y_{k+1}(a)=1|L_0=l,Y_k(a)=0)=\theta_a\tag{H3}$$

for $a=0,1$, *all* l *and some constants* θ_0,θ_1, *then*

$$\frac{\dfrac{\mathbb{E}\big[I(A_0=1)|L_0,Y_K=1\big]}{\mathbb{E}\big[I(A_0=0)|L_0,Y_K=1\big]}}{\dfrac{\mathbb{E}\big[I(A_0=1)\sum_{k=0}^{K-1}S_k|L_0\big]}{\mathbb{E}\big[I(A_0=0)\sum_{k=0}^{K-1}S_k|L_0\big]}}=\frac{\Pr(Y_{k+1}(1)=1|L_0,Y_k(1)=0)}{\Pr(Y_{k+1}(0)=1|L_0,Y_k(0)=0)}.$$

The proof to Theorem 5 is similar to that of Theorem 4 and therefore omitted.

Per-protocol effect

In this subsection, an individual qualifies as a case if and only if $Y_K = 1$ and the subject adheres to the protocol that was assigned at baseline. For any study participant, let S_k denote selection as a control for the period $[t_k, t_{k+1})$ and suppose S_k satisfies

$$\left.\begin{array}{c} S_k = 1 \Rightarrow Y_k = 0 \text{ with probability 1, and} \\ \Pr(S_k = 1|\overline{L}_k, \overline{A}_k, Y_k = 0) = \Pr(S_k = 1|\overline{A}_{k-1}, Y_k = 0) \text{ and} \\ \Pr(S_k = 1|\overline{A}_{k-1}, A_0 = ... = A_{k-1}, Y_k = 0) = \delta, \end{array}\right\} \tag{S4}$$

for some $\delta \in (0, 1]$.

Remark (Remark to Theorem 6) *Condition S4 holds if, for some constant δ_k^*,*

$$\left.\begin{array}{c} \Pr(S_k = 1) = \delta_k^* \Pr(Y_{k+1} = 1, Y_k = 0, \forall j < k : A_j = A_0) \textit{ and} \\ S_k \perp\!\!\!\perp (\overline{L}_k, \overline{A}_k, \overline{Y}_k)|(Y_k = 0, \forall j < k : A_j = A_0) \textit{ and} \\ S_k = 1 \Rightarrow (Y_k = 0, \forall j < k : A_j = A_0) \textit{ with probability 1.} \end{array}\right\} \tag{S4*}$$

The first requirement of S4* essentially means that the frequency of protocol-adherent incident cases in the kth window is proportional to the frequency of controls selected in this window. Under S4*, S4 is met with $\delta = \delta_k^* \Pr(Y_{k+1} = 1|Y_k = 0, \forall j < k : A_j = A_0)$, because

$$\begin{aligned} &\Pr(S_k = 1|\overline{L}_k, \overline{A}_k, \overline{Y}_k) \\ &\quad= \Pr(S_k = 1|Y_k = 0, \forall j < k : A_j = A_0) \times (1 - Y_k) \times I(\forall j < k : A_j = A_0) \\ &\quad= \frac{\Pr(S_k = 1)}{\Pr(Y_k = 0, \forall j < k : A_j = A_0)} \times (1 - Y_k) \times I(\forall j < k : A_j = A_0) \\ &\quad= \frac{\delta_k^* \Pr(Y_{k+1} = 1, Y_k = 0, \forall j < k : A_j = A_0)}{\Pr(Y_k = 0, \forall j < k : A_j = A_0)} \times (1 - Y_k) \times I(\forall j < k : A_j = A_0) \\ &\quad= \delta_k^* \Pr(Y_{k+1} = 1|Y_k = 0, \forall j < k : A_j = A_0) \times (1 - Y_k) \times I(\forall j < k : A_j = A_0). \end{aligned}$$

Similarly, condition S4 holds if, for some constant δ_k^{**},

$$\left.\begin{array}{c} \Pr(S_k = 1) = \delta_k^{**} \Pr(Y_{k+1} = 1, Y_k = 0) \textit{ and} \\ S_k \perp\!\!\!\perp (\overline{L}_k, \overline{A}_k, \overline{Y}_k)|(Y_k = 0) \textit{ and} \\ S_k = 1 \Rightarrow Y_k = 0 \textit{ with probability 1,} \end{array}\right\} \tag{S4**}$$

in which case, $\delta = \delta_k^{**} \Pr(Y_{k+1} = 1|Y_k = 0)$, because

$$\begin{aligned} &\Pr(S_k = 1|\overline{L}_k, \overline{A}_k, \overline{Y}_k) \\ &\quad= \Pr(S_k = 1|Y_k = 0) \times (1 - Y_k) \\ &\quad= \frac{\Pr(S_k = 1)}{\Pr(Y_k = 0)} \times (1 - Y_k) \\ &\quad= \frac{\delta_k^{**} \Pr(Y_{k+1} = 1, Y_k = 0)}{\Pr(Y_k = 0)} \times (1 - Y_k) \\ &\quad= \delta_k^{**} \Pr(Y_{k+1} = 1|Y_k = 0) \times (1 - Y_k). \end{aligned}$$

Theorem 6 (Risk-set sampling for marginal per-protocol effect) *Suppose SCE and S4 hold. If*

$$\Pr(Y_{k+1}(\overline{a}) = 1 | Y_k(\overline{a}) = 0) = \theta_a \tag{H4}$$

for $a = 0, 1$ *and some constants* θ_0, θ_1*, then*

$$\begin{aligned}
&\frac{\mathbb{E}\big[\sum_{k=0}^{K-1} I(A_k = 1) W_k I(Y_{k+1} = 1, Y_k = 0) | Y_K = 1, (\forall j : Y_j = 0 \Rightarrow A_j = A_0)\big]}{\mathbb{E}\big[\sum_{k=0}^{K-1} I(A_k = 0) W_k I(Y_{k+1} = 1, Y_k = 0) | Y_K = 1, (\forall j : Y_j = 0 \Rightarrow A_j = A_0)\big]} \\
&\qquad \frac{\mathbb{E}\big[I(A_0 = 1) \sum_{k=0}^{K-1} W_k S_k | \forall j : Y_j = 0 \Rightarrow A_j = A_0\big]}{\mathbb{E}\big[I(A_0 = 0) \sum_{k=0}^{K-1} W_k S_k | \forall j : Y_j = 0 \Rightarrow A_j = A_0\big]} \\
&\quad = \frac{\Pr(Y_{k+1}(\overline{1}) = 1 | Y_k(\overline{1}) = 0)}{\Pr(Y_{k+1}(\overline{0}) = 1 | Y_k(\overline{0}) = 0)},
\end{aligned}$$

where

$$W_k = \prod_{j=0}^{k} \frac{1}{\Pr(A_j = a_j | \overline{L}_j, \overline{A}_{j-1}, Y_j = 0, S_j = 1)}\bigg|_{a_j = A_j}.$$

Proof First, observe that $\Pr(A_k = a' | \overline{L}_k, (\forall j < k : A_j = a), Y_k = 0, S_k = 1) = \Pr(A_k = a' | \overline{L}_k, (\forall j < k : A_j = a), Y_k = 0)$ for $a', a = 0, 1$, because

$$\begin{aligned}
&\Pr(A_k = a' | \overline{L}_k, (\forall j < k : A_j = a), Y_k = 0, S_k = 1) \\
&\quad = \frac{\Pr(S_k = 1 | \overline{L}_k, (\forall j < k : A_j = a), A_k = a', Y_k = 0) \Pr(A_k = a' | \overline{L}_k, (\forall j < k : A_j = a), Y_k = 0)}{\Pr(S_k = 1 | \overline{L}_k, (\forall j < k : A_j = a), Y_k = 0)} \\
&\quad = \frac{\delta}{\delta} \Pr(A_k = a' | \overline{L}_k, (\forall j < k : A_j = a), Y_k = 0). \qquad \text{(by S4)}
\end{aligned}$$

Hence, if $\forall j < k : A_j = A_0$, then

$$W_k = \prod_{j=0}^{k} \frac{1}{\Pr(A_j = a_j | \overline{L}_j, \overline{A}_{j-1}, Y_j = 0)}\bigg|_{a_j = A_j}.$$

For the numerator of the main result of Theorem 6, we thus have

$$\begin{aligned}
&\frac{\mathbb{E}\big[\sum_{k=0}^{K-1} I(A_k = 1) W_k I(Y_{k+1} = 1, Y_k = 0) | Y_K = 1, (\forall j : Y_j = 0 \Rightarrow A_j = A_0)\big]}{\mathbb{E}\big[\sum_{k=0}^{K-1} I(A_k = 0) W_k I(Y_{k+1} = 1, Y_k = 0) | Y_K = 1, (\forall j : Y_j = 0 \Rightarrow A_j = A_0)\big]} \\
&\qquad \frac{\mathbb{E}\big[\sum_{k=0}^{K-1} I(A_k = a) W_k I(Y_{k+1} = 1, Y_k = 0, \forall j \le k : A_j = A_0)\big]}{\mathbb{E}\big[\sum_{k=0}^{K-1} I(A_k = a') W_k I(Y_{k+1} = 1, Y_k = 0, \forall j \le k : A_j = A_0)\big]} \\
&\quad = \frac{\sum_{k=0}^{K-1} \mathbb{E}\big[W_k Y_{k+1}(1 - Y_k) I(\forall j \le k : A_j = a)\big]}{\sum_{k=0}^{K-1} \mathbb{E}\big[W_k Y_{k+1}(1 - Y_k) I(\forall j \le k : A_j = a')\big]} \\
&\quad = \frac{\displaystyle\sum_{k=0}^{K-1} \sum_{\bar{l}_k} \frac{\Pr(Y_{k+1} = 1, Y_k = 0, \forall j \le k : A_j = a, \overline{L}_k = \bar{l}_k)}{\prod_{j=0}^{k} \Pr(A_j = a | Y_j = 0, \overline{L}_k = \bar{l}_k, \forall i < j : A_i = a)}}{\displaystyle\sum_{k=0}^{K-1} \sum_{\bar{l}_k} \frac{\Pr(Y_{k+1} = 1, Y_k = 0, \forall j \le k : A_j = a', \overline{L}_k = \bar{l}_k)}{\prod_{j=0}^{k} \Pr(A_j = a' | Y_j = 0, \overline{L}_k = \bar{l}_k, \forall i < j : A_i = a')}},
\end{aligned}$$

where

$$
\begin{aligned}
&\sum_{\bar{l}_k} \frac{\Pr(Y_{k+1}=1, Y_k=0, \forall j \le k: A_j = a, \overline{L}_k = \bar{l}_k)}{\prod_{j=0}^{k} \Pr(A_j = a | Y_j = 0, \overline{L}_k = \bar{l}_k, \forall i < j : A_i = a)} \\
&\quad = \sum_{\bar{l}_k} \Pr(Y_{k+1} = 1 | Y_k = 0, \overline{L}_k = \bar{l}_k, \forall j \le k : A_j = a) \\
&\qquad \times \Pr(L_k = l_k | Y_k = 0, \overline{L}_{k-1} = \bar{l}_{k-1}, \forall j < k : A_j = a) \\
&\qquad \times \prod_{j=0}^{k-1} \Pr(Y_{j+1} = 1 | Y_j = 0, \overline{L}_j = \bar{l}_j, \forall i \le j : A_i = a) \\
&\qquad \times \Pr(L_j = l_j | Y_j = 0, \overline{L}_{j-1} = \bar{l}_{j-1}, \forall i < j : A_i = a) \\
&\quad = \sum_{\bar{l}_k} \Pr(Y_{k+1}(\overline{a}) = 1 | Y_k(\overline{a}) = 0, \overline{L}_k = \bar{l}_k, \forall j \le k : A_j = a) \\
&\qquad \times \Pr(L_k = l_k | Y_k(\overline{a}) = 0, \overline{L}_{k-1} = \bar{l}_{k-1}, \forall j < k : A_j = a) \\
&\qquad \times \prod_{j=0}^{k-1} \Pr(Y_{j+1}(\overline{a}) = 1 | Y_j(\overline{a}) = 0, \overline{L}_j = \bar{l}_j, \forall i \le j : A_i = a) \\
&\qquad \times \Pr(L_j = l_j | Y_j(\overline{a}) = 0, \overline{L}_{j-1} = \bar{l}_{j-1}, \forall i < j : A_i = a) \\
&\qquad\qquad\qquad\qquad\qquad\qquad\qquad\qquad\qquad\qquad\qquad\qquad \text{(by consistency)} \\
&\quad = \sum_{\bar{l}_k} \Pr(Y_{k+1}(\overline{a}) = 1 | Y_k(\overline{a}) = 0, \overline{L}_k = \bar{l}_k, \forall j < k : A_j = a) \\
&\qquad \times \Pr(L_k = l_k | Y_k(\overline{a}) = 0, \overline{L}_{k-1} = \bar{l}_{k-1}, \forall j < k : A_j = a) \\
&\qquad \times \prod_{j=0}^{k-1} \Pr(Y_{j+1}(\overline{a}) = 1 | Y_j(\overline{a}) = 0, \overline{L}_j = \bar{l}_j, \forall i < j : A_i = a) \\
&\qquad \times \Pr(L_j = l_j | Y_j(\overline{a}) = 0, \overline{L}_{j-1} = \bar{l}_{j-1}, \forall i < j : A_i = a) \\
&\qquad\qquad\qquad\qquad\qquad\qquad\qquad \text{(by sequential conditional exchangeability)} \\
&\quad = \sum_{\bar{l}_{k-1}} \Pr(Y_{k+1}(\overline{a}) = 1 | Y_k(\overline{a}) = 0, \overline{L}_{k-1} = \bar{l}_{k-1}, \forall j < k : A_j = a) \\
&\qquad \times \prod_{j=0}^{k-1} \Pr(Y_{j+1}(\overline{a}) = 1 | Y_j(\overline{a}) = 0, \overline{L}_j = \bar{l}_j, \forall i < j : A_i = a) \\
&\qquad \times \Pr(L_j = l_j | Y_j(\overline{a}) = 0, \overline{L}_{j-1} = \bar{l}_{j-1}, \forall i < j : A_i = a) \\
&\quad = \sum_{\bar{l}_{k-1}} \Pr(Y_{k+1}(\overline{a}) = 1, Y_k(\overline{a}) = 0 | Y_{k-1}(\overline{a}) = 0, \overline{L}_{k-1} = \bar{l}_{k-1}, \forall j < k : A_j = a) \\
&\qquad \times \Pr(L_{k-1} = l_{k-1} | Y_{k-1}(\overline{a}) = 0, \overline{L}_{k-2} = \bar{l}_{k-2}, \forall j < k-1 : A_j = a) \\
&\qquad \times \prod_{j=0}^{k-2} \Pr(Y_{j+1}(\overline{a}) = 1 | Y_j(\overline{a}) = 0, \overline{L}_j = \bar{l}_j, \forall i < j : A_i = a) \\
&\qquad \times \Pr(L_j = l_j | Y_j(\overline{a}) = 0, \overline{L}_{j-1} = \bar{l}_{j-1}, \forall i < j : A_i = a) \\
&\quad \vdots \\
&\text{(by repeating previous three steps, under sequential conditional exchangeability)}
\end{aligned}
$$

$$= \Pr(Y_{k+1}(\overline{a}) = 1, Y_k(\overline{a}) = 0)$$

and, similarly,

$$\sum_{\bar{l}_k} \frac{\Pr(Y_{k+1} = 1, Y_k = 0, \forall j \leq k : A_j = a', \overline{L}_k = \bar{l}_k)}{\prod_{j=0}^{k} \Pr(A_j = a' | Y_j = 0, \overline{L}_k = \bar{l}_k, \forall i < j : A_i = a')} = \Pr(Y_{k+1}(\overline{a}') = 1, Y_k(\overline{a}') = 0).$$

Hence,

$$\begin{aligned}
&\frac{\mathbb{E}\big[\sum_{k=0}^{K-1} I(A_k = a) W_k I(Y_{k+1} = 1, Y_k = 0, \forall j \leq k : A_j = A_0)\big]}{\mathbb{E}\big[\sum_{k=0}^{K-1} I(A_k = a') W_k I(Y_{k+1} = 1, Y_k = 0, \forall j \leq k : A_j = A_0)\big]} \\
&\quad = \frac{\sum_{k=0}^{K-1} \Pr(Y_{k+1}(\overline{a}) = 1, Y_k(\overline{a}) = 0)}{\sum_{k=0}^{K-1} \Pr(Y_{k+1}(\overline{a}') = 1, Y_k(\overline{a}') = 0)} \\
&\quad = \frac{\sum_{k=0}^{K-1} \Pr(Y_{k+1}(\overline{a}) = 1 | Y_k(\overline{a}) = 0) \prod_{j=1}^{k} \Pr(Y_j(\overline{a}) = 0 | Y_{j-1}(\overline{a}) = 0)}{\sum_{k=0}^{K-1} \Pr(Y_{k+1}(\overline{a}') = 1 | Y_k(\overline{a}') = 0) \prod_{j=1}^{k} \Pr(Y_j(\overline{a}') = 0 | Y_{j-1}(\overline{a}') = 0)} \\
&\quad = \frac{\sum_{k=0}^{K-1} \theta_a (1 - \theta_a)^k}{\sum_{k=0}^{K-1} \theta_{a'} (1 - \theta_{a'})^k} \qquad \text{(H4)} \\
&\quad = \frac{1 - (1 - \theta_a)^K}{1 - (1 - \theta_{a'})^K} \\
&\qquad\qquad \text{(since } (1 - r) \textstyle\sum_{k=l}^{u} ar^k = a(r^l - r^{u+1}) \text{ for any real } a, r\text{)}
\end{aligned}$$

For the denominator, we have

$$\begin{aligned}
&\frac{\mathbb{E}\big[I(A_0 = a) \sum_{k=0}^{K-1} W_k S_k | \forall j : Y_j = 0 \Rightarrow A_j = A_0\big]}{\mathbb{E}\big[I(A_0 = a') \sum_{k=0}^{K-1} W_k S_k | \forall j : Y_j = 0 \Rightarrow A_j = A_0\big]} \\
&\quad = \frac{\mathbb{E}\big[\sum_{k=0}^{K-1} I(A_k = a) W_k S_k | \forall j : Y_j = 0 \Rightarrow A_j = A_0\big]}{\mathbb{E}\big[\sum_{k=0}^{K-1} I(A_k = a') W_k S_k | \forall j : Y_j = 0 \Rightarrow A_j = A_0\big]} \\
&\quad = \frac{\sum_{k=0}^{K-1} \mathbb{E}\big[I(A_k = a) W_k S_k | \forall j : Y_j = 0 \Rightarrow A_j = A_0\big]}{\sum_{k=0}^{K-1} \mathbb{E}\big[I(A_k = a') W_k S_k | \forall j : Y_j = 0 \Rightarrow A_j = A_0\big]} \\
&\quad = \frac{\sum_{k=0}^{K-1} \mathbb{E}\big[I(A_k = a) W_k S_k | Y_k = 0, \forall j \leq k : A_j = A_0\big] \Pr(Y_k = 0 | \forall j : Y_j = 0 \Rightarrow A_j = A_0)}{\sum_{k=0}^{K-1} \mathbb{E}\big[I(A_k = a') W_k S_k | Y_k = 0, \forall j \leq k : A_j = A_0\big] \Pr(Y_k = 0 | \forall j : Y_j = 0 \Rightarrow A_j = A_0)} \\
&\qquad\qquad \text{(by S4)} \\
&\quad = \frac{\sum_{k=0}^{K-1} \mathbb{E}\big[I(A_k = a) W_k S_k | Y_k = 0, \forall j \leq k : A_j = A_0\big] \Pr(Y_k = 0, \forall j \leq k : A_j = A_0)}{\sum_{k=0}^{K-1} \mathbb{E}\big[I(A_k = a') W_k S_k | Y_k = 0, \forall j \leq k : A_j = A_0\big] \Pr(Y_k = 0, \forall j \leq k : A_j = A_0)} \\
&\quad = \frac{\sum_{k=0}^{K-1} \mathbb{E}\big[W_k S_k | Y_k = 0, \forall j \leq k : A_j = a\big] \Pr(Y_k = 0, \forall j \leq k : A_j = a)}{\sum_{k=0}^{K-1} \mathbb{E}\big[W_k S_k | Y_k = 0, \forall j \leq k : A_j = a'\big] \Pr(Y_k = 0, \forall j \leq k : A_j = a')} \\
&\quad = \frac{\displaystyle\sum_{k=0}^{K-1} \sum_{\bar{l}_k} \frac{\mathbb{E}\big[S_k | Y_k = 0, \overline{L}_k = \bar{l}_k, \forall j \leq k : A_j = a\big] \Pr(Y_k = 0, \overline{L}_k = \bar{l}_k, \forall j \leq k : A_j = a)}{\prod_{j=0}^{k} \Pr(A_j = a | Y_j = 0, \overline{L}_j = \bar{l}_j, \forall i < j : A_i = a)}}{\displaystyle\sum_{k=0}^{K-1} \sum_{\bar{l}_k} \frac{\mathbb{E}\big[S_k | Y_k = 0, \overline{L}_k = \bar{l}_k, \forall j \leq k : A_j = a'\big] \Pr(Y_k = 0, \overline{L}_k = \bar{l}_k, \forall j \leq k : A_j = a')}{\prod_{j=0}^{k} \Pr(A_j = a' | Y_j = 0, \overline{L}_j = \bar{l}_j, \forall i < j : A_i = a')}}
\end{aligned}$$

$$
= \frac{\displaystyle\sum_{k=0}^{K-1}\sum_{\bar{l}_k} \delta \frac{\Pr(Y_k = 0, \overline{L}_k = \bar{l}_k, \forall j \leq k : A_j = a)}{\prod_{j=0}^{k} \Pr(A_j = a | Y_j = 0, \overline{L}_j = \bar{l}_j, \forall i < j : A_i = a)}}{\displaystyle\sum_{k=0}^{K-1}\sum_{\bar{l}_k} \delta \frac{\Pr(Y_k = 0, \overline{L}_k = \bar{l}_k, \forall j \leq k : A_j = a')}{\prod_{j=0}^{k} \Pr(A_j = a' | Y_j = 0, \overline{L}_j = \bar{l}_j, \forall i < j : A_i = a')}} \quad \text{(by S4)}
$$

$$
= \frac{\sum_{k=0}^{K-1}\sum_{\bar{l}_k} \delta \prod_{j=0}^{k} \Pr(Y_j = 0, L_j = l_j | Y_{j-1} = 0, \overline{L}_{j-1} = \bar{l}_{j-1}, \forall i < j : A_i = a)}{\sum_{k=0}^{K-1}\sum_{\bar{l}_k} \delta \prod_{j=0}^{k} \Pr(Y_j = 0, L_j = l_j | Y_{j-1} = 0, \overline{L}_{j-1} = \bar{l}_{j-1}, \forall i < j : A_i = a')}
$$

$$
= \frac{\displaystyle\sum_{k=0}^{K-1}\sum_{\bar{l}_k} \delta \prod_{j=0}^{k} \begin{array}{l} \Pr(L_j = l_j | Y_j = 0, \overline{L}_{j-1} = \bar{l}_{j-1}, \forall i < j : A_i = a) \times \\ \quad \Pr(Y_j = 0 | Y_{j-1} = 0, \overline{L}_{j-1} = \bar{l}_{j-1}, \forall i < j : A_i = a) \end{array}}{\displaystyle\sum_{k=0}^{K} -1 \sum_{\bar{l}_k} \delta \prod_{j=0}^{k} \begin{array}{l} \Pr(L_j = l_j | Y_j = 0, \overline{L}_{j-1} = \bar{l}_{j-1}, \forall i < j : A_i = a') \times \\ \quad \Pr(Y_j = 0 | Y_{j-1} = 0, \overline{L}_{j-1} = \bar{l}_{j-1}, \forall i < j : A_i = a') \end{array}}
$$

$$
= \frac{\displaystyle\sum_{k=0}^{K-1}\sum_{\bar{l}_k} \delta \prod_{j=0}^{k} \begin{array}{l} \Pr(L_j = l_j | Y_j(\overline{a}) = 0, \overline{L}_{j-1} = \bar{l}_{j-1}, \forall i < j : A_i = a) \times \\ \quad \Pr(Y_j(\overline{a}) = 0 | Y_{j-1}(\overline{a}) = 0, \overline{L}_{j-1} = \bar{l}_{j-1}, \forall i < j : A_i = a) \end{array}}{\displaystyle\sum_{k=0}^{K-1}\sum_{\bar{l}_k} \delta \prod_{j=0}^{k} \begin{array}{l} \Pr(L_j = l_j | Y_j(\overline{a}') = 0, \overline{L}_{j-1} = \bar{l}_{j-1}, \forall i < j : A_i = a') \times \\ \quad \Pr(Y_j(\overline{a}') = 0 | Y_{j-1}(\overline{a}') = 0, \overline{L}_{j-1} = \bar{l}_{j-1}, \forall i < j : A_i = a') \end{array}}
$$

(by consistency)

$$
= \frac{\displaystyle\sum_{k=0}^{K-1}\sum_{\bar{l}_{k-1}} \delta \prod_{j=0}^{k} \begin{array}{l} \Pr(Y_j(\overline{a}) = 0 | Y_{j-1}(\overline{a}) = 0, \overline{L}_{j-1} = \bar{l}_{j-1}, \forall i < j : A_i = a) \times \\ \quad \Pr(L_{j-1} = l_{j-1} | Y_{j-1}(\overline{a}) = 0, \overline{L}_{j-2} = \bar{l}_{j-2}, \forall i < j-1 : A_i = a) \end{array}}{\displaystyle\sum_{k=0}^{K-1}\sum_{\bar{l}_{k-1}} \delta \prod_{j=0}^{k} \begin{array}{l} \Pr(Y_j(\overline{a}') = 0 | Y_{j-1}(\overline{a}') = 0, \overline{L}_{j-1} = \bar{l}_{j-1}, \forall i < j : A_i = a') \times \\ \quad \Pr(L_{j-1} = l_{j-1} | Y_{j-1}(\overline{a}') = 0, \overline{L}_{j-2} = \bar{l}_{j-2}, \forall i < j-1 : A_i = a') \end{array}}
$$

$$
= \frac{\displaystyle\sum_{k=0}^{K-1}\sum_{\bar{l}_{k-1}} \delta \prod_{j=0}^{k} \begin{array}{l} \Pr(Y_j(\overline{a}) = 0 | Y_{j-1}(\overline{a}) = 0, \overline{L}_{j-1} = \bar{l}_{j-1}, \forall i < j-1 : A_i = a) \times \\ \quad \Pr(L_{j-1} = l_{j-1} | Y_{j-1}(\overline{a}) = 0, \overline{L}_{j-2} = \bar{l}_{j-2}, \forall i < j-1 : A_i = a) \end{array}}{\displaystyle\sum_{k=0}^{K-1}\sum_{\bar{l}_{k-1}} \delta \prod_{j=0}^{k} \begin{array}{l} \Pr(Y_j(\overline{a}') = 0 | Y_{j-1}(\overline{a}') = 0, \overline{L}_{j-1} = \bar{l}_{j-1}, \forall i < j-1 : A_i = a') \times \\ \quad \Pr(L_{j-1} = l_{j-1} | Y_{j-1}(\overline{a}') = 0, \overline{L}_{j-2} = \bar{l}_{j-2}, \forall i < j-1 : A_i = a') \end{array}}
$$

(by sequential conditional exchangeability)

$$
= \frac{\sum_{k=0}^{K-1}\sum_{\bar{l}_{k-1}} \delta \prod_{j=0}^{k} \Pr(Y_j(\overline{a}) = 0, L_{j-1} = l_{j-1} | Y_{j-1}(\overline{a}) = 0, \overline{L}_{j-2} = \bar{l}_{j-2}, \forall i < j-1 : A_i = a)}{\sum_{k=0}^{K-1}\sum_{\bar{l}_{k-1}} \delta \prod_{j=0}^{k} \Pr(Y_j(\overline{a}') = 0, L_{j-1} = l_{j-1} | Y_{j-1}(\overline{a}') = 0, \overline{L}_{j-2} = \bar{l}_{j-2}, \forall i < j-1 : A_i = a')}
$$

$$
= \frac{\displaystyle\sum_{k=0}^{K-1}\sum_{\bar{l}_{k-2}} \delta \begin{array}{l} \Pr(Y_k(\overline{a}) = 0 | Y_{k-1}(\overline{a}) = 0, \overline{L}_{k-2} = \bar{l}_{k-2}, \forall i < k-1 : A_i = a) \times \\ \quad \prod_{j=0}^{k-1} \Pr(Y_j(\overline{a}) = 0, L_{j-1} = l_{j-1} | Y_{j-1}(\overline{a}) = 0, \overline{L}_{j-2} = \bar{l}_{j-2}, \forall i < j-1 : A_i = a) \end{array}}{\displaystyle\sum_{k=0}^{K-1}\sum_{\bar{l}_{k-2}} \delta \begin{array}{l} \Pr(Y_k(\overline{a}') = 0 | Y_{k-1}(\overline{a}') = 0, \overline{L}_{k-2} = \bar{l}_{k-2}, \forall i < k-1 : A_i = a') \times \\ \quad \prod_{j=0}^{k-1} \Pr(Y_j(\overline{a}') = 0, L_{j-1} = l_{j-1} | Y_{j-1}(\overline{a}') = 0, \overline{L}_{j-2} = \bar{l}_{j-2}, \forall i < j-1 : A_i = a') \end{array}}
$$

$\vdots$ (by sequential conditional exchangeability)

$$
= \frac{\sum_{k=0}^{K-1} \delta \Pr(Y_k(\overline{a}) = 0)}{\sum_{k=0}^{K-1} \delta \Pr(Y_k(\overline{a}') = 0)}
$$

$$
= \frac{\sum_{k=0}^{K-1} \Pr(Y_k(\overline{a}) = 0)}{\sum_{k=0}^{K-1} \Pr(Y_k(\overline{a}') = 0)}
$$

$$
\begin{aligned}
&= \frac{1 + \sum_{k=1}^{K-1} \prod_{j=1}^{k} \Pr(Y_j(\overline{a}) = 0 | Y_{j-1}(\overline{a}) = 0)}{1 + \sum_{k=1}^{K-1} \prod_{j=1}^{k} \Pr(Y_j(\overline{a}') = 0 | Y_{j-1}(\overline{a}') = 0)} \\
&= \frac{1 + \sum_{k=1}^{K-1}(1-\theta_a)^k}{1 + \sum_{k=1}^{K}(1-\theta_{a'})^k} && \text{(by H4)} \\
&= \frac{1 + [1-\theta_a - (1-\theta_a)^{K-1}]/\theta_a}{1 + [1-\theta_{a'} - (1-\theta_{a'})^{K-1}]/\theta_{a'}} \\
& \qquad \text{(since } (1-r)\textstyle\sum_{k=l}^{u} ar^k = a(r^l - r^{u+1}) \text{ for any real } a, r) \\
&= \frac{\theta_{a'}(1-(1-\theta_a)^{K-1})}{\theta_a(1-(1-\theta_{a'})^{K-1})}.
\end{aligned}
$$

Hence,

$$
\begin{aligned}
&\frac{\mathbb{E}\big[\sum_{k=0}^{K-1} I(A_k = a) W_k I(Y_{k+1} = 1, Y_k = 0, \forall j \le k : A_j = A_0) | Y_K = 1\big]}{\mathbb{E}\big[\sum_{k=0}^{K-1} I(A_k = 1-a) W_k I(Y_{k+1} = 1, Y_k = 0, \forall j \le k : A_j = A_0) | Y_K = 1\big]} \\
&\qquad \bigg/ \frac{\mathbb{E}\big[\sum_{k=0}^{K-1} I(A_k = a) W_k S_k\big]}{\mathbb{E}\big[\sum_{k=0}^{K-1} I(A_k = 1-a) W_k S_k\big]} \\
&= \frac{1-(1-\theta_a)^{K-1}}{1-(1-\theta_{a'})^{K-1}} \times \frac{\theta_a(1-(1-\theta_{a'})^{K-1})}{\theta_{a'}(1-(1-\theta_a)^{K-1})} \\
&= \theta_a / \theta_{a'},
\end{aligned}
$$

which completes the proof. □

Appendix C: Identification results for exact 1:M matching strategies

Intention-to-treat effect

In this subsection, cases are defined by $Y_K = 1$ and have baseline exposure A_0. All cases are assigned a (possibly variable) number $M \ge 0$ of control exposures A'_i, $i = 1, ..., M$, subject to

$$
\left.
\begin{array}{c}
\Pr(M > 0 | Y_K = 1) > 0 \text{ and} \\
M \perp\!\!\!\perp A_0 | (L_0, Y_K = 1) \text{ and} \\
\forall l, a, a' : \Pr(A'_i = a' | L_0 = l, A_0 = a, Y_K = 1, M, M > 0) = \Pr(A_0 = a' | L_0 = l),
\end{array}
\right\} \tag{M1}
$$

or

$$
\left.
\begin{array}{c}
\Pr(M > 0 | Y_K = 1) > 0 \text{ and} \\
M \perp\!\!\!\perp A_0 | (L_0, Y_K = 1) \text{ and} \\
\forall l, a, a' : \Pr(A'_i = a' | L_0 = l, A_0 = a, Y_K = 1, M, M > 0) = \Pr(A_0 = a' | L_0 = l, Y_K = 0),
\end{array}
\right\} \tag{M2}
$$

or

$$\left.\begin{array}{r}\Pr(M>0|Y_K=1)>0 \text{ and}\\ M \perp\!\!\!\perp A_0|(L_0,Y_K=1,J) \text{ and}\\ \forall l,a,a': \Pr(A_i'=a'|L_0=l,A_0=a,\overline{Y}_K,J=j,M,M>0)=\Pr(A_0=a'|L_0=l,Y_j=0), \quad \text{where}\\ J=\max\{k=0,1,...,K:Y_k=0\}.\end{array}\right\} \tag{M3}$$

That is, cases are matched with subjects that have the same baseline covariate level and who are alive at baseline (M1), at the end of study (M2), or whenever the case is alive (M3).

For simplicity, it is assumed below that the variables are discrete. The results can however be extended to more general distributions.

Theorem 7 (Case-base sampling for marginal intention-to-treat effect) *If M1 and BCE hold and*

$$\frac{\Pr(Y_K=1|L_0=l,A_0=1)}{\Pr(Y_K=1|L_0=l,A_0=0)}=\theta \tag{H1}$$

for all l and some constant θ, then

$$\frac{\mathbb{E}\big[\sum_{i=1}^{M} I(A_i'=0,A_0=1)\big|Y_K=1,M>0\big]}{\mathbb{E}\big[\sum_{i=1}^{M} I(A_i'=1,A_0=0)\big|Y_K=1,M>0\big]}=\frac{\Pr(Y_K(1)=1)}{\Pr(Y_K(0)=1)}.$$

Proof We have

$$\frac{\mathbb{E}\big[\sum_{i=1}^{M} I(A_i'=0,A_0=1)\big|Y_K=1,M>0\big]}{\mathbb{E}\big[\sum_{i=1}^{M} I(A_i'=1,A_0=0)\big|Y_K=1,M>0\big]}=\frac{\mathbb{E}\big[\sum_{i=1}^{M} I(A_i'=0)\big|A_0=1,Y_K=1,M>0\big]}{\mathbb{E}\big[\sum_{i=1}^{M} I(A_i'=1)\big|A_0=0,Y_K=1,M>0\big]}$$
$$\times \text{Odds}(A_0=1|Y_K=1,M>0),$$

where

$$\frac{\mathbb{E}\big[\sum_{i=1}^{M} I(A_i'=0)\big|A_0=1,Y_K=1,M>0\big]}{\mathbb{E}\big[\sum_{i=1}^{M} I(A_i'=1)\big|A_0=0,Y_K=1,M>0\big]}$$
$$=\frac{\sum_{m>0}\mathbb{E}\big[\sum_{i=1}^{m} I(A_i'=0)\big|A_0=1,Y_K=1,M=m\big]\Pr(M=m|A_0=1,Y_K=1,M>0)}{\sum_{m>0}\mathbb{E}\big[\sum_{i=1}^{m} I(A_i'=1)\big|A_0=0,Y_K=1,M=m\big]\Pr(M=m|A_0=0,Y_K=1,M>0)}$$
$$=\frac{\sum_{m>0}\sum_{i=1}^{m}\sum_l \Pr(A_i'=0|L_0=l,A_0=1,Y_K=1,M=m)\Pr(M=m,L_0=l|A_0=1,Y_K=1,M> \text{[illegible]}}{\sum_{m>0}\sum_{i=1}^{m}\sum_l \Pr(A_i'=1|L_0=l,A_0=0,Y_K=1,M=m)\Pr(M=m,L_0=l|A_0=0,Y_K=1,M> \text{[illegible]}}$$
$$=\frac{\sum_{m>0}\sum_{i=1}^{m}\sum_l \Pr(A_0=0|L_0=l)\Pr(M=m,L_0=l|A_0=1,Y_K=1,M>0)}{\sum_{m>0}\sum_{i=1}^{m}\sum_l \Pr(A_0=1|L_0=l)\Pr(M=m,L_0=l|A_0=0,Y_K=1,M>0)} \quad \text{(by M1)}$$
$$=\frac{\sum_{m>0}\sum_{i=1}^{m}\sum_l \Pr(A_0=0|L_0=l)\Pr(M=m,L_0=l,A_0=1|Y_K=1)}{\sum_{m>0}\sum_{i=1}^{m}\sum_l \Pr(A_0=1|L_0=l)\Pr(M=m,L_0=l,A_0=0|Y_K=1)}\frac{1}{\text{Odds}(A_0=1|Y_K=1,M>0)}$$
$$=\frac{\sum_{m>0}\sum_{i=1}^{m}\sum_l q(l,m)\Pr(Y_K=1|L_0=l,A_0=1)}{\sum_{m>0}\sum_{i=1}^{m}\sum_l q(l,m)\Pr(Y_K=1|L_0=l,A_0=0)}$$

$$\times \frac{1}{\text{Odds}(A_0 = 1|Y_K = 1, M > 0)}$$

(under M1 and definition of $q(l,m)$ (see below))

$$= \frac{\sum_{m>0}\sum_{i=1}^{m}\sum_l q(l,m)\theta \Pr(Y_K = 1|L_0 = l, A_0 = 0)}{\sum_{m>0}\sum_{i=1}^{m}\sum_l q(l,m) \Pr(Y_K = 1|L_0 = l, A_0 = 0)} \frac{1}{\text{Odds}(A_0 = 1|Y_K = 1, M > 0)}$$

(by H1)

$$= \frac{\theta}{\text{Odds}(A_0 = 1|Y_K = 1, M > 0)}$$

where $q(l,m) = \Pr(M = m|L_0 = l, Y_K = 1)\Pr(A_0 = 0|L_0 = l)\Pr(A_0 = 1|L_0 = l)\Pr(L_0 = l)$.

It follows that

$$\frac{\mathbb{E}\big[\sum_{i=1}^{M} I(A_i' = 0, A_0 = 1)\big|Y_K = 1, M > 0\big]}{\mathbb{E}\big[\sum_{i=1}^{M} I(A_i' = 1, A_0 = 0)\big|Y_K = 1, M > 0\big]} = \frac{\Pr(Y_K = 1|L_0, A_0 = 1)}{\Pr(Y_K = 1|L_0, A_0 = 0)}$$

$$= \frac{\Pr(Y_K(1) = 1|L_0, A_0 = 1)}{\Pr(Y_K(0) = 1|L_0, A_0 = 0)}$$

(by consistency)

$$= \frac{\Pr(Y_K(1) = 1|L_0)}{\Pr(Y_K(0) = 1|L_0)}$$

(by baseline conditional exchangeability)

$$= \frac{\Pr(Y_K(1) = 1)}{\Pr(Y_K(0) = 1)}.$$

□

Theorem 8 (Survivor sampling for conditional intention-to-treat effect) *Suppose M2 and BCE hold. If*

$$\frac{\text{Odds}(Y_K = 1|L_0, A_0 = 1)}{\text{Odds}(Y_K = 1|L_0, A_0 = 0)} = \theta \tag{H5}$$

for some constant θ, *then*

$$\frac{\mathbb{E}\big[\sum_{i=1}^{M} I(A_i' = 0, A_0 = 1)\big|Y_K = 1, M > 0\big]}{\mathbb{E}\big[\sum_{i=1}^{M} I(A_i' = 1, A_0 = 0)\big|Y_K = 1, M > 0\big]} = \frac{\text{Odds}(Y_K(1) = 1|L_0)}{\text{Odds}(Y_K(0) = 1|L_0)}.$$

Proof We have

$$\frac{\mathbb{E}\big[\sum_{i=1}^{M} I(A_i' = 0, A_0 = 1)\big|Y_K = 1, M > 0\big]}{\mathbb{E}\big[\sum_{i=1}^{M} I(A_i' = 1, A_0 = 0)\big|Y_K = 1, M > 0\big]} = \frac{\mathbb{E}\big[\sum_{i=1}^{M} I(A_i' = 0)\big|A_0 = 1, Y_K = 1, M > 0\big]}{\mathbb{E}\big[\sum_{i=1}^{M} I(A_i' = 1)\big|A_0 = 0, Y_K = 1, M > 0\big]} \times \text{Odds}(A_0 = 1|Y_K = 1, M > 0),$$

where

$$\frac{\mathbb{E}\big[\sum_{i=1}^{M} I(A_i' = 0)\big|A_0 = 1, Y_K = 1, M > 0\big]}{\mathbb{E}\big[\sum_{i=1}^{M} I(A_i' = 1)\big|A_0 = 0, Y_K = 1, M > 0\big]}$$

$$= \frac{\sum_{m>0} \mathbb{E}\big[\sum_{i=1}^{m} I(A'_i = 0)\big|A_0 = 1, Y_K = 1, M = m\big] \Pr(M = m|A_0 = 1, Y_K = 1)}{\sum_{m>0} \mathbb{E}\big[\sum_{i=1}^{m} I(A'_i = 1)\big|A_0 = 0, Y_K = 1, M = m\big] \Pr(M = m|A_0 = 0, Y_K = 1)}$$

$$= \frac{\sum_{m>0} \sum_{i=1}^{m} \sum_l \Pr(A'_i = 0|L_0 = l, A_0 = 1, Y_K = 1, M = m) \Pr(M = m, L_0 = l|A_0 = 1, Y_K = 1, M >}{\sum_{m>0} \sum_{i=1}^{m} \sum_l \Pr(A'_i = 1|L_0 = l, A_0 = 0, Y_K = 1, M = m) \Pr(M = m, L_0 = l|A_0 = 0, Y_K = 1, M >}$$

$$= \frac{\sum_{m>0} \sum_{i=1}^{m} \sum_l \Pr(A_0 = 0|L_0 = l, Y_K = 0) \Pr(M = m, L_0 = l|A_0 = 1, Y_K = 1, M > 0)}{\sum_{m>0} \sum_{i=1}^{m} \sum_l \Pr(A_0 = 1|L_0 = l, Y_K = 0) \Pr(M = m, L_0 = l|A_0 = 0, Y_K = 1, M > 0)} \quad \text{(by M2)}$$

$$= \frac{\displaystyle\sum_{m>0} \sum_{i=1}^{m} \sum_l \frac{\Pr(Y_K = 0|L_0 = 0, A_0 = 0) \Pr(A_0 = 0|L_0 = l)}{\Pr(Y_K = 0|L_0 = l)} \Pr(M = m, L_0 = l, A_0 = 1|Y_K = 1)}{\displaystyle\sum_{m>0} \sum_{i=1}^{m} \sum_l \frac{\Pr(Y_K = 0|L_0 = 0, A_0 = 1) \Pr(A_0 = 1|L_0 = l)}{\Pr(Y_K = 0|L_0 = l)} \Pr(M = m, L_0 = l, A_0 = 0|Y_K = 1)} \times \frac{1}{\text{Odds}(A_0 = 1|Y_K = 1, M > 0)}$$

$$= \frac{\sum_{m>0} \sum_{i=1}^{m} \sum_l q(l, m) \Pr(Y_K = 1|L_0 = l, A_0 = 1) \Pr(Y_K = 0|L_0 = 0, A_0 = 0)}{\sum_{m>0} \sum_{i=1}^{m} \sum_l q(l, m) \Pr(Y_K = 1|L_0 = l, A_0 = 0) \Pr(Y_K = 0|L_0 = 0, A_0 = 1)} \times \frac{1}{\text{Odds}(A_0 = 1|Y_K = 1, M > 0)}$$

$$\text{(under M2 and definition of } q(l, m) \text{ (see below))}$$

$$= \frac{\sum_{m>0} \sum_{i=1}^{m} \sum_l q(l, m)\theta \Pr(Y_K = 1|L_0 = l, A_0 = 0) \Pr(Y_K = 0|L_0 = 0, A_0 = 1)}{\sum_{m>0} \sum_{i=1}^{m} \sum_l q(l, m) \Pr(Y_K = 1|L_0 = l, A_0 = 0) \Pr(Y_K = 0|L_0 = 0, A_0 = 1)} \times \frac{1}{\text{Odds}(A_0 = 1|Y_K = 1, M > 0)} \quad \text{(by H5)}$$

$$= \frac{\theta}{\text{Odds}(A_0 = 1|Y_K = 1, M > 0)}$$

where $q(l, m) = \Pr(M = m|L_0 = l, Y_K = 1) \Pr(A_0 = 0|L_0 = l) \Pr(A_0 = 1|L_0 = l) \Pr(L_0 = l)/\Pr(Y_K = 0|L_0 = l)$.

From the definition of θ, it follows that

$$\frac{\mathbb{E}\big[\sum_{i=1}^{M} I(A'_i = 0, A_0 = 1)\big|Y_K = 1, M > 0\big]}{\mathbb{E}\big[\sum_{i=1}^{M} I(A'_i = 1, A_0 = 0)\big|Y_K = 1, M > 0\big]} = \frac{\text{Odds}(Y_K(1) = 1|L_0, A_0 = 1)}{\text{Odds}(Y_K(0) = 1|L_0, A_0 = 0)} \quad \text{(by consistency)}$$

$$= \frac{\text{Odds}(Y_K(1) = 1|L_0)}{\text{Odds}(Y_K(0) = 1|L_0)} \quad \text{(by baseline conditional exchangeability)}$$

$$= \frac{\text{Odds}(Y_K(1) = 1)}{\text{Odds}(Y_K(0) = 1)}.$$

□

Theorem 9 (Risk-set sampling for conditional intention-to-treat effect) *Suppose M3 and BCE hold. If*

$$\frac{\Pr(Y_{j+1} = 1|L_0, A_0 = 1, Y_j = 0)}{\Pr(Y_{j+1} = 1|L_0, A_0 = 0, Y_j = 0)} = \theta \qquad \text{(H6)}$$

for $j = 0, 1, ..., K$ and some constant θ, then

$$\frac{\mathbb{E}\big[\sum_{i=1}^{M} I(A_i' = 0, A_0 = 1)\big|Y_K = 1, M > 0\big]}{\mathbb{E}\big[\sum_{i=1}^{M} I(A_i' = 1, A_0 = 0)\big|Y_K = 1, M > 0\big]} = \frac{\Pr(Y_{j+1}(1) = 1|L_0, Y_j(1) = 0)}{\Pr(Y_{j+1}(0) = 1|L_0, Y_j(0) = 0)}.$$

Proof If $J = \max\{k = 0, 1, ..., K : Y_k = 0\}$, then

$$\begin{aligned}
&\frac{\mathbb{E}\big[\sum_{i=1}^{M} I(A_i' = 0, A_0 = 1)\big|Y_K = 1, M > 0\big]}{\mathbb{E}\big[\sum_{i=1}^{M} I(A_i' = 1, A_0 = 0)\big|Y_K = 1, M > 0\big]} \\
&\quad = \frac{\sum_{m>0} \mathbb{E}\big[\sum_{i=1}^{m} I(A_i' = 0, A_0 = 1)\big|Y_K = 1, M = m\big] \Pr(M = m|Y_K = 1, M > 0)}{\sum_{m>0} \mathbb{E}\big[\sum_{i=1}^{m} I(A_i' = 1, A_0 = 0)\big|Y_K = 1, M = m\big] \Pr(M = m|Y_K = 1, M > 0)} \\
&\quad = \frac{\sum_{m>0} \mathbb{E}\big[\sum_{i=1}^{m} I(A_i' = 0, A_0 = 1)\big|Y_K = 1, M = m\big] \Pr(M = m, Y_K = 1)}{\sum_{m>0} \mathbb{E}\big[\sum_{i=1}^{m} I(A_i' = 1, A_0 = 0)\big|Y_K = 1, M = m\big] \Pr(M = m, Y_K = 1)} \\
&\quad = \frac{\sum_{m>0} \sum_{j=0}^{K-1} \sum_l \mathbb{E}\big[\sum_{i=1}^{m} I(A_i' = 0, A_0 = 1)\big|L_0 = l, J = j, M = m\big] \Pr(L_0 = l, J = j, M = m)}{\sum_{m>0} \sum_{j=0}^{K-1} \sum_l \mathbb{E}\big[\sum_{i=1}^{m} I(A_i' = 1, A_0 = 0)\big|L_0 = l, J = j, M = m\big] \Pr(L_0 = l, J = j, M = m)} \\
&\quad = \frac{\sum_{m>0} \sum_{i=1}^{m} \sum_{j=0}^{K-1} \sum_l \mathbb{E}\big[I(A_i' = 0, A_0 = 1)\big|L_0 = l, J = j, M = m\big] \Pr(L_0 = l, J = j, M = m)}{\sum_{m>0} \sum_{i=1}^{m} \sum_{j=0}^{K-1} \sum_l \mathbb{E}\big[I(A_i' = 1, A_0 = 0)\big|L_0 = l, J = j, M = m\big] \Pr(L_0 = l, J = j, M = m)} \\
&\quad = \frac{\displaystyle\sum_{m>0} \sum_{i=1}^{m} \sum_{j=0}^{K-1} \sum_l \begin{matrix} \mathbb{E}\big[I(A_i' = 0, A_0 = 1)\big|L_0 = l, Y_j = 0, Y_{j+1} = 1, M = m\big] \\ \times \Pr(L_0 = l, Y_j = 0, Y_{j+1} = 1, M = m) \end{matrix}}{\displaystyle\sum_{m>0} \sum_{i=1}^{m} \sum_{j=0}^{K-1} \sum_l \begin{matrix} \mathbb{E}\big[I(A_i' = 1, A_0 = 0)\big|L_0 = l, Y_j = 0, Y_{j+1} = 1, M = m\big] \\ \times \Pr(L_0 = l, Y_j = 0, Y_{j+1} = 1, M = m) \end{matrix}} \\
&\quad = \frac{\displaystyle\sum_{m>0} \sum_{i=1}^{m} \sum_{j=0}^{K-1} \sum_l \begin{matrix} \Pr(A_i' = 0|L_0 = l, A_0 = 1, Y_j = 0, Y_{j+1} = 1, M = m) \\ \times \Pr(L_0 = l, A_0 = 1, Y_j = 0, Y_{j+1} = 1, M = m) \end{matrix}}{\displaystyle\sum_{m>0} \sum_{i=1}^{m} \sum_{j=0}^{K-1} \sum_l \begin{matrix} \Pr(A_i' = 1|L_0 = l, A_0 = 0, Y_j = 0, Y_{j+1} = 1, M = m) \\ \times \Pr(L_0 = l, A_0 = 0, Y_j = 0, Y_{j+1} = 1, M = m) \end{matrix}} \\
&\quad = \frac{\sum_{m>0} \sum_{i=1}^{m} \sum_{j=0}^{K-1} \sum_l \Pr(A_0 = 0|L_0 = l, Y_j = 0) \Pr(L_0 = l, A_0 = 1, Y_j = 0, Y_{j+1} = 1, M = m)}{\sum_{m>0} \sum_{i=1}^{m} \sum_{j=0}^{K-1} \sum_l \Pr(A_0 = 1|L_0 = l, Y_j = 0) \Pr(L_0 = l, A_0 = 0, Y_j = 0, Y_{j+1} = 1, M = m)} \\
&\qquad\qquad \text{(by M3)} \\
&\quad = \frac{\sum_{m>0} \sum_{i=1}^{m} \sum_{j=0}^{K-1} \sum_l q_j(l, m) \Pr(Y_{j+1} = 1|L_0 = l, A_0 = 1, Y_j = 0)}{\sum_{m>0} \sum_{i=1}^{m} \sum_{j=0}^{K-1} \sum_l q_j(l, m) \Pr(Y_{j+1} = 1|L_0 = l, A_0 = 0, Y_j = 0)} \\
&\qquad\qquad \text{(under M3 and definition of } q_j(l, m) \text{ (see below))} \\
&\quad = \theta \frac{\sum_{m>0} \sum_{i=1}^{m} \sum_{j=0}^{K-1} \sum_l q_j(l, m) \Pr(Y_{j+1} = 1|L_0 = l, A_0 = 0, Y_j = 0)}{\sum_{m>0} \sum_{i=1}^{m} \sum_{j=0}^{K-1} \sum_l q_j(l, m) \Pr(Y_{j+1} = 1|L_0 = l, A_0 = 0, Y_j = 0)} \\
&\qquad\qquad \text{(by H6)} \\
&\quad = \theta.
\end{aligned}$$

where $q_j(l, m) = \Pr(M = m|L_0 = l, Y_j = 0) \Pr(A_0 = 1|L_0 = l, Y_j = 0) \Pr(A_0 = 0|L_0 = l, Y_j = 0) \Pr(L_0 = l, Y_j = 0)$.

Thus,

$$\frac{\mathbb{E}\big[\sum_{i=1}^{M} I(A_i' = 0, A_0 = 1)\big|Y_K = 1\big]}{\mathbb{E}\big[\sum_{i=1}^{M} I(A_i' = 1, A_0 = 0)\big|Y_K = 1\big]} = \frac{\Pr(Y_{j+1} = 1|L_0, A_0 = 1, Y_j = 0)}{\Pr(Y_{j+1} = 1|L_0, A_0 = 0, Y_j = 0)}$$

$$= \frac{\Pr(Y_{j+1}(1) = 1|L_0, A_0 = 1, Y_j(1) = 0)}{\Pr(Y_{j+1}(0) = 1|L_0, A_0 = 0, Y_j(0) = 0)}$$

(by consistency)

$$= \frac{\Pr(Y_{j+1}(1) = 1|L_0, Y_j(1) = 0)}{\Pr(Y_{j+1}(0) = 1|L_0, Y_j(0) = 0)}.$$

(by baseline conditional exchangeability)

□

Per-protocol effect

In this subsection, an individual qualifies as a case if and only if $Y_K = 1$ and the subject adheres to the protocol that was assigned at baseline (i.e., $A_k = A_0$ for all $k = 0, 1, ..., K$ if $Y_k = 0$). All cases are assigned a (possibly variable) number $M \geq 0$ control exposures A_i', $i = 1, ..., M$, subject to

$$\left.\begin{array}{c} \Pr(M > 0|Y_K = 1, \forall j : (Y_j = 0 \Rightarrow A_j = A_0)) > 0 \text{ and} \\ M \perp\!\!\!\perp A_0|(J, Y_K = 1, \overline{L}_J = \overline{l}_J, \forall i \leq J : A_i = A_0) \text{ and} \\ \forall \overline{l}, a : \Pr(A_i' = a'|\overline{L}_J = \overline{l}_J, \forall j \leq J : A_j = A_0, A_0 = a, Y_J = 0, J, M, M > 0) \\ = \Pr(A_J = a'|\overline{L}_J = \overline{l}_J, \forall j \leq J : A_j = A_0, Y_J = 0), \quad \text{where} \\ J = \max\{k = 0, 1, ..., K : Y_k = 0\}. \end{array}\right\} \tag{M4}$$

Theorem 10 (Risk-set sampling for conditional per-protocol effect) *Suppose M4 holds. If*

$$\frac{\Pr(Y_{j+1} = 1|\overline{L}_j = \overline{l}_j, Y_j = 0, \forall i \leq j : A_i = 1)}{\Pr(Y_{j+1} = 1|\overline{L}_j = \overline{l}_j, Y_j = 0, \forall i \leq j : A_i = 0)} = \theta \tag{H7}$$

for all $j, \overline{l}_j$ and some constant θ, then

$$\frac{\mathbb{E}\Big[\sum_{i=1}^{M} I(A_i' = 0, A_0 = 1)\Big|Y_K = 1, \forall j : (Y_j = 0 \Rightarrow A_j = A_0), M > 0\Big]}{\mathbb{E}\Big[\sum_{i=1}^{M} I(A_i' = 1, A_0 = 0)\Big|Y_K = 1, \forall j : (Y_j = 0 \Rightarrow A_j = A_0), M > 0\Big]}$$

$$= \frac{\Pr(Y_{j+1}(\overline{1}) = 1|\overline{L}_j = \overline{l}_j, Y_j(\overline{1}) = 0, \forall i \leq j : A_i = 1)}{\Pr(Y_{j+1}(\overline{0}) = 1|\overline{L}_j = \overline{l}_j, Y_j(\overline{0}) = 0, \forall i \leq j : A_i = 0)}.$$

Proof Let $J = \max\{k = 0, 1, ..., K : Y_k = 0\}$. Then, for $a = 0, 1$,

$$\mathbb{E}\left[\sum_{i=1}^{M} I(A_i' = 1 - a, A_0 = a)\middle|Y_K = 1, \forall j \leq J : A_j = A_0, M > 0\right]$$

$$= \sum_{j=0}^{K-1} \sum_{\overline{l}_j} \mathbb{E}\left[\sum_{i=1}^{M} I(A_i' = 1 - a, A_0 = a)\middle|\overline{L}_j = \overline{l}_j, J = j, Y_K = 1, \forall j \leq J : A_j = A_0, M > 0\right]$$

$$
\begin{aligned}
&\quad \times \Pr(\overline{L}_j = \bar{l}_j, J = j | Y_K = 1, \forall i \le J : A_i = A_0, M > 0) \\
&= \sum_{j=0}^{K-1} \sum_{\bar{l}_j} \mathbb{E}\Bigg[\sum_{i=1}^{M} I(A_i' = 1 - a, A_0 = a) \Bigg| \overline{L}_j = \bar{l}_j, Y_j = 0, Y_{j+1} = 1, \forall j \le J : A_j = A_0, M > 0 \Bigg] \\
&\quad \times \Pr(\overline{L}_j = \bar{l}_j, Y_j = 0, Y_{j+1} = 1 | Y_K = 1, \forall i \le J : A_i = A_0, M > 0) \\
&= \sum_{m>0} \sum_{j=0}^{K-1} \sum_{\bar{l}_j} \mathbb{E}\Bigg[\sum_{i=1}^{m} I(A_i' = 1 - a, A_0 = a) \Bigg| \overline{L}_j = \bar{l}_j, Y_j = 0, Y_{j+1} = 1, \forall j \le J : A_j = A_0, M = m \Bigg] \\
&\quad \times \Pr(M = m, \overline{L}_j = \bar{l}_j, Y_j = 0, Y_{j+1} = 1 | \forall j \le J : A_j = A_0, M > 0) \\
&= \sum_{m>0} \sum_{u=1}^{m} \sum_{j=0}^{K-1} \sum_{\bar{l}_j} \mathbb{E}\Bigg[I(A_u' = 1 - a, A_0 = a) \Bigg| \overline{L}_j = \bar{l}_j, Y_j = 0, Y_{j+1} = 1, \forall j \le J : A_j = A_0, M = m \Bigg] \\
&\quad \times \Pr(M = m, \overline{L}_j = \bar{l}_j, Y_j = 0, Y_{j+1} = 1 | \forall j \le J : A_j = A_0, M > 0) \\
&= \sum_{m>0} \sum_{u=1}^{m} \sum_{j=0}^{K-1} \sum_{\bar{l}_j} \Pr(A_u' = 1 - a | \overline{L}_j = \bar{l}_j, Y_j = 0, Y_{j+1} = 1, \forall j \le J : A_j = a, M = m) \\
&\quad \times \Pr(M = m, A_0 = a, \overline{L}_j = \bar{l}_j, Y_j = 0, Y_{j+1} = 1 | \forall j \le J : A_j = A_0, M > 0) \\
&= \sum_{m>0} \sum_{u=1}^{m} \sum_{j=0}^{K-1} \sum_{\bar{l}_j} \Pr(A_0 = 1 - a | Y_j = 0, \overline{L}_j = \bar{l}_j, \forall i \le j : A_i = A_0) \\
&\quad \times \Pr(M = m, A_0 = a, \overline{L}_j = \bar{l}_j, Y_j = 0, Y_{j+1} = 1 | \forall j \le J : A_j = A_0, M > 0) \\
&\qquad\qquad\qquad\qquad\qquad\qquad\qquad\qquad\qquad\qquad\qquad\qquad\qquad\qquad \text{(by M4)} \\
&= \sum_{m>0} \sum_{u=1}^{m} \sum_{j=0}^{K-1} \sum_{\bar{l}_j} \Pr(A_0 = 1 - a | Y_j = 0, \overline{L}_j = \bar{l}_j, \forall i \le j : A_i = A_0) \\
&\quad \times \Pr(M = m, \overline{L}_j = \bar{l}_j, A_0 = a, Y_{j+1} = 1, Y_j = 0, \forall i \le j : A_i = A_0) \\
&\quad \times \Pr(Y_K = 1, \forall i : (Y_i = 0 \Rightarrow A_i = A_0), M > 0)^{-1} \\
&= \sum_{m>0} \sum_{u=1}^{m} \sum_{j=0}^{K-1} \sum_{\bar{l}_j} \Pr(Y_{j+1} = 1 | \overline{L}_j = \bar{l}_j, A_0 = a, Y_j = 0, \forall i \le j : A_i = A_0) \\
&\quad \times q_j(\bar{l}_j, m) \Pr(Y_K = 1, \forall i : (Y_i = 0 \Rightarrow A_i = A_0), M > 0)^{-1}, \\
&\qquad\qquad\qquad\qquad\qquad\qquad\qquad\qquad\qquad\qquad\qquad\qquad\qquad \text{(under M4)}
\end{aligned}
$$

where

$$
\begin{aligned}
q_j(\bar{l}_j, m) = &\Pr(M = m | \overline{L}_j = \bar{l}_j, Y_j = 0, Y_{j+1} = 1, \forall i \le j : A_i = A_0) \\
&\times \Pr(A_0 = 1 - a | Y_j = 0, \overline{L}_j = \bar{l}_j, \forall i \le j : A_i = A_0) \\
&\times \Pr(A_0 = a | Y_j = 0, \overline{L}_j = \bar{l}_j, \forall i \le j : A_i = A_0) \\
&\times \Pr(\overline{L}_j = \bar{l}_j, Y_j = 0, \forall i \le j : A_i = A_0).
\end{aligned}
$$

It follows that

$$
\frac{\mathbb{E}\Big[\sum_{i=1}^{M} I(A_i' = 0, A_0 = 1) \Big| Y_K = 1, \forall j : (Y_j = 0 \Rightarrow A_j = A_0), M > 0 \Big]}{\mathbb{E}\Big[\sum_{i=1}^{M} I(A_i' = 1, A_0 = 0) \Big| Y_K = 1, \forall j : (Y_j = 0 \Rightarrow A_j = A_0), M > 0 \Big]}
$$

$$
\begin{aligned}
&= \frac{\displaystyle\sum_{m>0}\sum_{u=1}^{m}\sum_{j=0}^{K-1}\sum_{\bar{l}_j} \begin{array}{l}\Pr(Y_{j+1}=1|\overline{L}_j=\bar{l}_j, A_0=1, Y_j=0, \forall i\le j: A_i=A_0)\\ \quad\times q_j(\bar{l}_j,m)\Pr(Y_K=1,\forall i:(Y_i=0\Rightarrow A_i=A_0), M>0)^{-1}\end{array}}{\displaystyle\sum_{m>0}\sum_{u=1}^{m}\sum_{j=0}^{K-1}\sum_{\bar{l}_j} \begin{array}{l}\Pr(Y_{j+1}=1|\overline{L}_j=\bar{l}_j, A_0=0, Y_j=0, \forall i\le j: A_i=A_0)\\ \quad\times q_j(\bar{l}_j,m)\Pr(Y_K=1,\forall i:(Y_i=0\Rightarrow A_i=A_0), M>0)^{-1}\end{array}}\\
&= \frac{\sum_{m>0}\sum_{u=1}^{m}\sum_{j=0}^{K-1}\sum_{\bar{l}_j}\Pr(Y_{j+1}=1|\overline{L}_j=\bar{l}_j, A_0=1, Y_j=0,\forall i\le j: A_i=A_0)q_j(\bar{l}_j,m)}{\sum_{m>0}\sum_{u=1}^{m}\sum_{j=0}^{K-1}\sum_{\bar{l}_j}\Pr(Y_{j+1}=1|\overline{L}_j=\bar{l}_j, A_0=1, Y_j=0,\forall i\le j: A_i=A_0)q_j(\bar{l}_j,m)}\\
&= \theta\frac{\sum_{m>0}\sum_{u=1}^{m}\sum_{j=0}^{K-1}\sum_{\bar{l}_j}\Pr(Y_{j+1}=1|\overline{L}_j=\bar{l}_j Y_j=0,\forall i\le j: A_i=0)q_j(\bar{l}_j,m)}{\sum_{m>0}\sum_{u=1}^{m}\sum_{j=0}^{K-1}\sum_{\bar{l}_j}\Pr(Y_{j+1}=1|\overline{L}_j=\bar{l}_j, Y_j=0,\forall i\le j: A_i=0)q_j(\bar{l}_j,m)}\\
&\hspace{20em}\text{(by H7)}\\
&= \theta.
\end{aligned}
$$

The desired results follows by consistency. □

Appendix D: Parametric identification by conditional logistic regression for exact or partial 1:M matching

We now allow for the possibility that cases ($Y_K = 1$) are matched to $M \geq 0$ controls on only part of L_0. That part of L_0 on which exact matching is done will be denoted L_0^*; the other part is denoted L_0', so that $L_0 = (L_0^*, L_0')$. The identification result below rests on the assumption that cases are assigned $M \geq 0$ pairs (A_i', L_i') of baseline exposure and baseline covariate data, $i = 1, ..., M$, subject to

$$
\left.\begin{array}{r}
\Pr(M>0|Y_K=1)>0 \text{ and}\\
M \perp\!\!\!\perp (A_0, L_0)|(L_0^*, Y_K=1) \text{ and}\\
\forall l, l', a: \Pr(A_i'=a, L_i'=l'|L_0^*=l, L_0', A_0, Y_K=1, M, M>0) =\\
\Pr(A_0=a, L_0'=l'|L_0^*=l, Y_K=0) \text{ and}\\
(L_0', A_0), (L_1', A_1'), ..., (L_M', A_M') \text{ are mutually independent given } (L_0^*, Y_K=1, M>0).
\end{array}\right\} \tag{M2*}
$$

It is assumed below that the variables are discrete with finite support for simplicity. The results can however be extended to more general distributions.

Theorem 11 (Conditional logistic regression for conditional intention-to-treat effect) *Suppose BCE and M2* hold. For some known real-valued functions f_j, $j = 1, ..., p$, assume the following model:*

$$
\operatorname{logit}\Pr(Y_K(a)=1|L_0) = \alpha + \sum_{j=1}^{p} f_j(a, L_0^*, L_0')\beta_j \tag{Outcome Model}
$$

For $i = 0, ..., M$, let $X_{i,j} = f_j(A_i', L_0^*, L_i') - f_j(A_0, L_0^*, L_0')$, with $A_0' = A_0$, and assume for any $\gamma_1, ..., \gamma_p \in \mathbb{R}$, not all zero, that

$$
\Pr\left(\bigvee_{i=1}^{M}\left[\sum_{j=1}^{p}\gamma_j X_{i,j} \neq 0\right]\middle| Y_K=1, M>0\right) > 0, \tag{Linear Independence}
$$

where $\bigvee$ *denotes the logical OR operator (i.e., given any indexed collection* $(P_i)_{i\in I}$ *of propositions,* $\bigvee_{i\in I} P_i$ *is the proposition that* P_i *is true for at least one* $i \in I$*). Then,*

$$\mathbb{E}\left[-\log\left(1+\sum_{i=1}^{M}\exp\left[\sum_{j=1}^{p}X_{i,j}\tilde{\beta}_j\right]\right)^{-1}\middle|Y_K=1,M>0\right]$$

is uniquely maximized at $\tilde{\beta}=\beta$.

Proof We first demonstrate that

$$\mathbb{E}\left[-\log\left(1+\sum_{i=1}^{M}\exp\left[\sum_{j=1}^{p}X_{i,j}\tilde{\beta}_j\right]\right)^{-1}\middle|Y_K=1,M>0\right]$$

has at most one maximum by showing that it is strictly concave as a function of $\tilde{\beta}$. Let $X=(X_1,...,X_M)$ and $X_i=(X_{i,1},...,X_{i,p})$, $i=1,...,M$. To show that function f,

$$\begin{aligned}f(\beta)&=\mathbb{E}\left[\log\left(1+\sum_{i=1}^{M}\exp\left[\sum_{j=1}^{p}X_{i,j}\beta_j\right]\right)^{-1}\middle|Y_K=1,M>0\right]\\&=\sum_{m>0}\sum_{x}\log\left(1+\sum_{i=1}^{m}\exp\left[\sum_{j=1}^{p}x_{i,j}\beta_j\right]\right)^{-1}\Pr(X=x|Y_K=1,M=m)\Pr(M=m|Y_K=1,M>0),\end{aligned}$$

is convex (and $-f$ concave) it suffices to show that its Hessian is positive semidefinite, i.e., that $\sum_{t=1}^{p}\sum_{u=1}^{p}\beta_k\beta_l H_{k,l}(\beta)\geq 0$ for all $\beta\in\mathbb{R}^p$, where

$$H_{k,l}(\beta)=\frac{\partial}{\partial\beta_l}\frac{\partial}{\partial\beta_k}f(\beta).$$

Positive definiteness of the Hessian, i.e., $\sum_{k=1}^{p}\sum_{l=1}^{p}\beta_k\beta_l H_{k,l}(\beta)>0$ for all $\beta\in\mathbb{R}^p$ such that $\beta_k\neq 0$ for some $k\in\{1,...,p\}$, implies strict convexity of f (and $-f$ strictly concave).

Letting $g(X_i,\beta)=\exp\left\{\sum_{j=1}^{p}X_{i,j}\beta_j\right\}$ for $i=1,...,M$, we have

$$\begin{aligned}H_{k,l}(\beta)&=\frac{\partial}{\partial\beta_l}\frac{\partial}{\partial\beta_k}f(\beta)\\&=\frac{\partial}{\partial\beta_l}\sum_{m>0}\sum_{x}\frac{\sum_{i=1}^{m}x_{i,k}g(x_i,\beta)}{1+\sum_{i=1}^{m}g(x_i,\beta)}\Pr(X=x|Y_K=1,M=m)\Pr(M=m|Y_K=1,M>0)\\&=\frac{\partial}{\partial\beta_l}\sum_{m>0}\sum_{x}\frac{\sum_{i=1}^{m}x_{i,k}g(x_i,\beta)}{1+\sum_{i=1}^{m}g(x_i,\beta)}\Pr(X=x|Y_K=1,M=m)\Pr(M=m|Y_K=1,M>0)\\&=\sum_{m>0}\sum_{x}\left(1+\sum_{i=1}^{m}g(x_i,\beta)\right)^{-2}\left[\left(1+\sum_{i=1}^{m}g(x_i,\beta)\right)\left(\sum_{i=1}^{m}X_{i,k}X_{i,l}g(x_i,\beta)\right)\right.\\&\qquad\left.-\left(\sum_{i=1}^{m}X_{i,k}g(x_i,\beta)\right)\left(\sum_{i=1}^{m}X_{i,l}g(x_i,\beta)\right)\right]\end{aligned}$$

$$\times \Pr(X = x | Y_K = 1, M = m) \Pr(M = m | Y_K = 1, M > 0),$$

so that, with $v_i = \sqrt{g(x_i, \beta)}$ and $w_i = \sum_{j=1}^{p} x_{i,j} \beta_j \sqrt{g(x_i, \beta)}$,

$$\begin{aligned}
&\sum_{k=1}^{p} \sum_{l=1}^{p} \beta_k \beta_l H_{k,l}(\beta) \\
&= \sum_{m>0} \sum_{x} \frac{\Pr(X = x | Y_K = 1, M = m) \Pr(M = m | Y_K = 1, M > 0)}{\left(1 + \sum_{i=1}^{m} g(x_i, \beta)\right)^2} \\
&\quad \times \Bigg[\sum_{k=1}^{p} \sum_{l=1}^{p} \beta_k \beta_l \left(1 + \sum_{i=1}^{m} g(x_i, \beta)\right) \left(\sum_{i=1}^{m} x_{i,k} x_{i,l} g(x_i, \beta)\right) \\
&\quad - \sum_{k=1}^{p} \sum_{l=1}^{p} \beta_k \beta_l \left(\sum_{i=1}^{m} x_{i,k} g(x_i, \beta)\right) \left(\sum_{i=1}^{m} x_{i,l} g(x_i, \beta)\right) \Bigg] \\
&= \sum_{m>0} \sum_{x} \frac{\Pr(X = x | Y_K = 1, M = m) \Pr(M = m | Y_K = 1, M > 0)}{\left(1 + \sum_{i=1}^{m} g(x_i, \beta)\right)^2} \\
&\quad \times \Bigg[\left(1 + \sum_{i=1}^{m} g(x_i, \beta)\right) \left(\sum_{i=1}^{m} g(x_i, \beta) \left(\sum_{k=1}^{p} \beta_k x_{i,k}\right) \left(\sum_{l=1}^{p} \beta_l x_{i,l}\right)\right) \\
&\quad - \left(\sum_{i=1}^{m} \sum_{k=1}^{p} \beta_k x_{i,k} g(x_i, \beta)\right) \left(\sum_{i=1}^{m} \sum_{l=1}^{p} \beta_l x_{i,l} g(x_i, \beta)\right) \Bigg] \\
&= \sum_{m>0} \sum_{x} \frac{\Pr(X = x | Y_K = 1, M = m) \Pr(M = m | Y_K = 1, M > 0)}{\left(1 + \sum_{i=1}^{m} g(x_i, \beta)\right)^2} \\
&\quad \times \left[\left(1 + \sum_{i=1}^{m} g(x_i, \beta)\right) \left(\sum_{i=1}^{m} \left(\sum_{k=1}^{p} \beta_k x_{i,k} \sqrt{g(x_i, \beta)}\right)^2\right) - \left(\sum_{i=1}^{m} \sum_{k=1}^{p} \beta_k x_{i,k} g(x_i, \beta)\right)^2 \right] \\
&= \sum_{m>0} \sum_{x} \frac{\Pr(X = x | Y_K = 1, M = m) \Pr(M = m | Y_K = 1, M > 0)}{\left(1 + \sum_{i=1}^{m} g(x_i, \beta)\right)^2} \\
&\quad \times \left[\sum_{i=1}^{m} \left(\sum_{k=1}^{p} \beta_k x_{i,k} \sqrt{g(x_i, \beta)}\right)^2 + \left(\sum_{i=1}^{m} v_{i,j}^2\right) \left(\sum_{i=1}^{m} w_{i,j}^2\right) - \left(\sum_{i=1}^{m} v_{i,j} v_{i,j}\right)^2 \right] \\
&\geq \sum_{m>0} \sum_{x} \frac{\Pr(X = x | Y_K = 1, M = m) \Pr(M = m | Y_K = 1, M > 0)}{\left(1 + \sum_{i=1}^{m} g(x_i, \beta)\right)^2} \sum_{i=1}^{m} \left(\sum_{k=1}^{p} \beta_k x_{i,k} \sqrt{g(x_i, \beta)}\right)^2. \\
&\qquad \text{(by the Cauchy-Schwarz inequality)}
\end{aligned}$$

Now,

$$\begin{aligned}
&\sum_{m>0} \sum_{x} \frac{\Pr(X = x | Y_K = 1, M = m) \Pr(M = m | Y_K = 1, M > 0)}{\left(1 + \sum_{i=1}^{m} g(x_i, \beta)\right)^2} \sum_{i=1}^{m} \left(\sum_{k=1}^{p} \beta_k x_{i,k} \sqrt{g(x_i, \beta)}\right)^2 \\
&= \sum_{m>0} \sum_{x} \frac{\Pr(X = x | Y_K = 1, M = m) \Pr(M = m | Y_K = 1, M > 0)}{\left(1 + \sum_{i=1}^{m} g(x_i, \beta)\right)^2} \sum_{i=1}^{m} g(x_i, \beta) \left(\sum_{k=1}^{p} \beta_k x_{i,k}\right)^2 \\
&= \mathbb{E}\left[\left(1 + \sum_{i=1}^{M} g(X_i, \beta)\right)^{-2} \sum_{i=1}^{M} g(X_i, \beta) \left(\sum_{k=1}^{p} \beta_k X_{i,k}\right)^2 \middle| Y_K = 1, M > 0 \right] \\
&\geq 0
\end{aligned}$$

with strict inequality under Linear Independence. Thus,

$$\mathbb{E}\Bigg[-\log\left(1+\sum_{i=1}^{M}\exp\left[\sum_{j=1}^{p}X_{i,j}\tilde{\beta}_j\right]\right)^{-1}\Bigg|Y_K=1, M>0\Bigg]$$

has at most one maximum.

It remains to be shown that

$$\mathbb{E}\Bigg[-\log\left(1+\sum_{i=1}^{M}\exp\left[\sum_{j=1}^{p}X_{i,j}\tilde{\beta}_j\right]\right)^{-1}\Bigg|Y_K=1, M>0\Bigg]$$

is maximized at $\tilde{\beta}=\beta$, i.e., $\partial/\partial\tilde{\beta}_k f(\tilde{\beta})=0$ for all $k=1,...,p$ at $\tilde{\beta}=\beta$.

Now,

$$\begin{aligned}\frac{\partial}{\partial\tilde{\beta}_k}f(\tilde{\beta}) &= \mathbb{E}\Bigg[\frac{\sum_{i=1}^{M}X_{i,k}g(X_i,\tilde{\beta})}{1+\sum_{i=1}^{m}g(X_i,\tilde{\beta})}\Bigg|Y_K=1,M>0\Bigg]\\ &= \sum_{l^*}\sum_{m>0}\mathbb{E}\Bigg[\frac{\sum_{i=1}^{m}X_{i,k}g(X_i,\tilde{\beta})}{1+\sum_{i=1}^{m}g(X_i,\tilde{\beta})}\Bigg|L_0^*=l^*,Y_K=1,M=m\Bigg]\Pr(L_0^*=l^*,M=m|,Y_K=1,M>0),\end{aligned}$$

where

$$\begin{aligned}&\mathbb{E}\Bigg[\frac{\sum_{i=1}^{m}X_{i,k}g(X_i,\tilde{\beta})}{1+\sum_{i=1}^{m}g(X_i,\tilde{\beta})}\Bigg|L_0^*=l^*,Y_K=1,M=m\Bigg]\\ &\quad=\sum_{l_0,...,l_m}\sum_{a_0,...,a_m}\frac{\sum_{i=1}^{m}[f_k(a_i,l^*,l_i)-f_k(a_0,l^*,l_0)]\exp\big\{\sum_{k=1}^{p}[f_k(a_i,l^*,l_i)-f_k(a_0,l^*,l_0)]\tilde{\beta}_k\big\}}{1+\sum_{i=1}^{m}\exp\big\{\sum_{k=1}^{p}[f_k(a_i,l^*,l_i)-f_k(a_0,l^*,l_0)]\tilde{\beta}_k\big\}}\\ &\qquad\times\Pr(A_0=a_0,A_1'=a_1,...,A_m=a_m,L_0'=l_0,...,L_m'=l_m|L_0^*=l^*,Y_K=1,M=m)\\ &\quad=\sum_{l_0,...,l_m}\sum_{a_0,...,a_m}\frac{\sum_{i=1}^{m}[f_k(a_i,l^*,l_i)-f_k(a_0,l^*,l_0)]\exp\big\{\sum_{k=1}^{p}[f_k(a_i,l^*,l_i)-f_k(a_0,l^*,l_0)]\tilde{\beta}_k\big\}}{1+\sum_{i=1}^{m}\exp\big\{\sum_{k=1}^{p}[f_k(a_i,l^*,l_i)-f_k(a_0,l^*,l_0)]\tilde{\beta}_k\big\}}\\ &\times h(a_0,...,a_M,l_0,...,l_M)\Pr\Big(A_0=a_0,A_1'=a_1,...,A_M=a_M,L_0'=l_0,...,L_m'=l_m\Big|\\ &\bigvee_\sigma\big[(A_0=a_{\sigma(0)},L_0'=_{\sigma(0)},A_1'=a_{\sigma(1)},L_1'=_{\sigma(1)},...,A_m=a_{\sigma(m)},L_m'=_{\sigma(m)})\big],L_0^*=l^*,Y_K=1,M=m\Big),\end{aligned}$$

where permutation σ denotes a bijection from $\{0,1,...,M\}$ to itself and

$$\begin{aligned}&h(a_0,...,a_M,l_0,...,l_M)\\ &\quad=\Pr\Big(\bigvee_\sigma\big[(A_0=a_{\sigma(0)},L_0'=_{\sigma(0)},A_1'=a_{\sigma(1)},L_1'=_{\sigma(1)},...,A_m=a_{\sigma(m)},L_m'=_{\sigma(m)})\big]\Big|\\ &\qquad L_0^*=l^*,Y_K=1,M=m\Big).\end{aligned}$$

Next, let $B_0=(L_0',A_0)$ and $B_i=(L_i',A_i')$, $i=1,2,...,M$. Let $b_i=(l_i,a_i)$ for $i=0,...,M$. We have

$$\Pr\Bigg(B_0=b_0,,...,B_M=b_M\Bigg|\bigvee_\sigma\big[(B_0,...,B_M)=(b_{\sigma(0)},...,b_{\sigma(M)})\big],L_0^*,Y_K=1,M,M>0\Bigg)$$

$$= \frac{\Pr(B_0 = b_0, ..., B_M = b_M | L_0^*, Y_K = 1, M > 0)}{\Pr\left(\bigvee_\sigma \left[B_0 = b_{\sigma(0)}, ..., B_M = a_{\sigma(M)} \right] \middle| L_0^*, Y_K = 1, M, M > 0 \right)}$$

$$\propto \frac{\Pr(B_0 = b_0, ..., B_M = b_M | L_0^*, Y_K = 1, M > 0)}{\sum_\sigma \Pr\left(B_0 = b_{\sigma(0)}, ..., B_M = a_{\sigma(M)} \middle| L_0^*, Y_K = 1, M, M > 0 \right)}$$

$$= \frac{\prod_{i=0}^M \Pr(B_i = b_i | L_0^*, Y_K = 1, M, M > 0)}{\sum_\sigma \prod_{i=0}^M \Pr(B_i = b_{\sigma(i)} | L_0^*, Y_K = 1, M, M > 0)}$$

(by mutual independence of M2*)

$$= \frac{\Pr(B_0 = b_0 | L_0^*, Y_K = 1) \prod_{i=1}^M \Pr(B_0 = b_i | L_0^*, Y_K = 0)}{\sum_\sigma \Pr(B_0 = b_{\sigma(0)} | L_0^*, Y_K = 1) \prod_{i=1}^M \Pr(B_0 = b_{\sigma(i)} | L_0^*, Y_K = 0)}$$

(by M2*)

$$= \frac{\Pr(Y_K = 1 | B_0 = b_0, L_0^*) \prod_{i=1}^M [1 - \Pr(Y_K = 1 | B_0 = b_i, L_0^*)]}{\sum_\sigma \Pr(Y_K = 1 | B_0 = b_{\sigma(0)}, L_0^*) \prod_{i=1}^M [1 - \Pr(Y_K = 1 | B_0 = b_{\sigma(i)}, L_0^*)]}$$

$$= \frac{\Pr(Y_K = 1 | L_0 = (L_0^*, l_0), A_0 = a_0) \prod_{i=1}^M [1 - \Pr(Y_K = 1 | L_0 = (L_0^*, l_i), A_0 = a_i)]}{\sum_\sigma \Pr(Y_K = 1 | L_0 = (L_0^*, l_{\sigma(0)}), A_0 = a_{\sigma(0)}) \prod_{i=1}^M [1 - \Pr(Y_K = 1 | L_0 = (L_0^*, l_{\sigma(i)}), A_0 = a_{\sigma(i)})]}$$

$$= \frac{\dfrac{\Pr(Y_K = 1 | L_0 = (L_0^*, l_0), A_0 = a_0)}{1 - \Pr(Y_K = 1 | L_0 = (L_0^*, l_0), A_0 = a_0)} \displaystyle\prod_{i=0}^M [1 - \Pr(Y_K = 1 | L_0 = (L_0^*, l_i), A_0 = a_i)]}{\displaystyle\sum_\sigma \dfrac{\Pr(Y_K = 1 | L_0 = (L_0^*, l_{\sigma(0)}), A_0 = a_{\sigma(0)})}{1 - \Pr(Y_K = 1 | L_0 = (L_0^*, l_{\sigma(0)}), A_0 = a_{\sigma(0)})} \prod_{i=0}^M [1 - \Pr(Y_K = 1 | L_0 = (L_0^*, l_{\sigma(i)}), A_0 = a_{\sigma(i)})]}$$

$$= \frac{\dfrac{\Pr(Y_K = 1 | L_0 = (L_0^*, l_0), A_0 = a_0)}{1 - \Pr(Y_K = 1 | L_0 = (L_0^*, l_0), A_0 = a_0)}}{\displaystyle\sum_\sigma \dfrac{\Pr(Y_K = 1 | L_0 = (L_0^*, l_{\sigma(0)}), A_0 = a_{\sigma(0)})}{1 - \Pr(Y_K = 1 | L_0 = (L_0^*, l_{\sigma(0)}), A_0 = a_{\sigma(0)})}}$$

$$\propto \frac{\dfrac{\Pr(Y_K = 1 | L_0 = (L_0^*, l_0), A_0 = a_0)}{1 - \Pr(Y_K = 1 | L_0 = (L_0^*, l_0), A_0 = a_0)}}{\displaystyle\sum_{i=0}^M \dfrac{\Pr(Y_K = 1 | L_0 = (L_0^*, l_i), A_0 = a_i)}{1 - \Pr(Y_K = 1 | L_0 = (L_0^*, l_i), A_0 = a_i)}}$$

$$= \frac{\dfrac{\operatorname{expit}\left\{\alpha + \sum_{j=1}^p f_j(a_0, L_0^*, l_0)\beta_j\right\}}{1 - \operatorname{expit}\left\{\alpha + \sum_{j=1}^p f_j(a_0, L_0^*, l_0)\beta_j\right\}}}{\displaystyle\sum_{i=0}^M \dfrac{\operatorname{expit}\left\{\alpha + \sum_{j=1}^p f_j(a_i, L_0^*, l_i)\beta_j\right\}}{1 - \operatorname{expit}\left\{\alpha + \sum_{j=1}^p f_j(a_i, L_0^*, l_i)\beta_j\right\}}}$$

$$= \frac{\exp\left[\sum_{j=1}^p f_j(a_0, L_0^*, l_0)\beta_j\right]}{\sum_{i=0}^M \exp\left[\sum_{j=1}^p f_j(a_i, L_0^*, l_i)\beta_j\right]}$$

$$= \left(\sum_{i=0}^M \exp\left[\sum_{j=1}^p \left[f_j(a_i, L_0^*, l_i) - f_j(a_0, L_0^*, l_0) \right] \beta_j \right] \right)^{-1}$$

$$= \left(1 + \sum_{i=1}^M \exp\left[\sum_{j=1}^p \left[f_j(a_i, L_0^*, l_i) - f_j(a_0, L_0^*, l_0) \right] \beta_j \right] \right)^{-1}.$$

Thus,

$$\mathbb{E}\left[\frac{\sum_{i=1}^m X_{i,j} g(X_i, \tilde{\beta})}{1 + \sum_{i=1}^m g(X_i, \tilde{\beta})} \middle| L_0^* = l^*, Y_K = 1, M = m \right]$$

$$
\begin{aligned}
&\propto \sum_{l_0,\ldots,l_m} \sum_{a_0,\ldots,a_m} \frac{\sum_{i=1}^{m}[f_k(a_i,l^*,l_i)-f_k(a_0,l^*,l_0)]\exp\left\{\sum_{k=1}^{p}[f_k(a_i,l^*,l_i)-f_k(a_0,l^*,l_0)]\tilde{\beta}_k\right\}}{1+\sum_{i=1}^{m}\exp\left\{\sum_{k=1}^{p}[f_k(a_i,l^*,l_i)-f_k(a_0,l^*,l_0)]\tilde{\beta}_k\right\}} \\
&\quad \times \frac{1}{1+\sum_{i=1}^{m}\exp\left\{\sum_{k=1}^{p}[f_k(a_i,l^*,l_i)-f_k(a_0,l^*,l_0)]\beta_k\right\}} h(a_0,...,a_M,l_0,...,l_M) \\
&\propto \sum_{l_0,\ldots,l_m} \sum_{a_0,\ldots,a_m} h(a_0,...,a_M,l_0,...,l_M) \\
&\quad \times \sum_{i=1}^{m}[f_k(a_i,l^*,l_i)-f_k(a_0,l^*,l_0)] \frac{\exp\left\{\sum_{k=1}^{p}[f_k(a_i,l^*,l_i)-f_k(a_0,l^*,l_0)]\tilde{\beta}_k\right\}}{1+\sum_{i=1}^{m}\exp\left\{\sum_{k=1}^{p}[f_k(a_i,l^*,l_i)-f_k(a_0,l^*,l_0)]\tilde{\beta}_k\right\}} \\
&\quad \times \frac{1}{1+\sum_{i=1}^{m}\exp\left\{\sum_{k=1}^{p}[f_k(a_i,l^*,l_i)-f_k(a_0,l^*,l_0)]\beta_k\right\}} \\
&\propto \sum_{\{(l_0,a_0),\ldots,(l_m,a_M)\}} h(a_0,...,a_M,l_0,...,l_M) \\
&\quad \times \sum_{u=1}^{m}\sum_{i=1}^{m}[f_k(a_i,l^*,l_i)-f_k(a_u,l^*,l_u)] \frac{\exp\left\{\sum_{k=1}^{p}[f_k(a_i,l^*,l_i)-f_k(a_u,l^*,l_u)]\tilde{\beta}_k\right\}}{1+\sum_{i=1}^{m}\exp\left\{\sum_{k=1}^{p}[f_k(a_i,l^*,l_i)-f_k(a_u,l^*,l_u)]\tilde{\beta}_k\right\}} \\
&\quad \times \frac{1}{1+\sum_{i=1}^{m}\exp\left\{\sum_{k=1}^{p}[f_k(a_i,l^*,l_i)-f_k(a_u,l^*,l_u)]\beta_k\right\}} \\
&= \sum_{\{(l_0,a_0),\ldots,(l_m,a_M)\}} h(a_0,...,a_M,l_0,...,l_M) \\
&\quad \times \sum_{u=1}^{m}\sum_{i=1}^{m}[f_k(a_i,l^*,l_i)-f_k(a_u,l^*,l_u)] \frac{\exp\left\{\sum_{k=1}^{p}f_k(a_i,l^*,l_i)\tilde{\beta}_k\right\}}{\sum_{i=0}^{m}\exp\left\{\sum_{k=1}^{p}f_k(a_i,l^*,l_i)\tilde{\beta}_k\right\}} \\
&\quad \times \frac{\exp\left\{\sum_{k=1}^{p}f_k(a_u,l^*,l_u)\beta_k\right\}}{\sum_{i=0}^{m}\exp\left\{\sum_{k=1}^{p}f_k(a_i,l^*,l_i)\beta_k\right\}} \\
&= \sum_{\{(l_0,a_0),\ldots,(l_m,a_M)\}} h(a_0,...,a_M,l_0,...,l_M) \\
&\quad \times \Bigg[\sum_{u,i\in\{1,\ldots,m\}:i>u} [f_k(a_i,l^*,l_i)-f_k(a_u,l^*,l_u)] \frac{\exp\left\{\sum_{k=1}^{p}f_k(a_i,l^*,l_i)\tilde{\beta}_k\right\}}{\sum_{i=0}^{m}\exp\left\{\sum_{k=1}^{p}f_k(a_i,l^*,l_i)\tilde{\beta}_k\right\}} \\
&\quad \times \frac{\exp\left\{\sum_{k=1}^{p}f_k(a_u,l^*,l_u)\beta_k\right\}}{\sum_{i=0}^{m}\exp\left\{\sum_{k=1}^{p}f_k(a_i,l^*,l_i)\beta_k\right\}} \\
&\quad + \sum_{u,i\in\{1,\ldots,m\}:i<u} [f_k(a_i,l^*,l_i)-f_k(a_u,l^*,l_u)] \frac{\exp\left\{\sum_{k=1}^{p}f_k(a_i,l^*,l_i)\tilde{\beta}_k\right\}}{\sum_{i=0}^{m}\exp\left\{\sum_{k=1}^{p}f_k(a_i,l^*,l_i)\tilde{\beta}_k\right\}} \\
&\quad \times \frac{\exp\left\{\sum_{k=1}^{p}f_k(a_u,l^*,l_u)\beta_k\right\}}{\sum_{i=0}^{m}\exp\left\{\sum_{k=1}^{p}f_k(a_i,l^*,l_i)\beta_k\right\}} \Bigg] \\
&= \sum_{\{(l_0,a_0),\ldots,(l_m,a_M)\}} h(a_0,...,a_M,l_0,...,l_M) \\
&\quad \times \Bigg[\sum_{u,i\in\{1,\ldots,m\}:i>u} [f_k(a_i,l^*,l_i)-f_k(a_u,l^*,l_u)] \frac{\exp\left\{\sum_{k=1}^{p}f_k(a_i,l^*,l_i)\tilde{\beta}_k\right\}}{\sum_{i=0}^{m}\exp\left\{\sum_{k=1}^{p}f_k(a_i,l^*,l_i)\tilde{\beta}_k\right\}} \\
&\quad \times \frac{\exp\left\{\sum_{k=1}^{p}f_k(a_u,l^*,l_u)\beta_k\right\}}{\sum_{i=0}^{m}\exp\left\{\sum_{k=1}^{p}f_k(a_i,l^*,l_i)\beta_k\right\}} \\
&\quad - \sum_{u,i\in\{1,\ldots,m\}:i>u} [f_k(a_i,l^*,l_i)-f_k(a_u,l^*,l_u)] \frac{\exp\left\{\sum_{k=1}^{p}f_k(a_u,l^*,l_u)\tilde{\beta}_k\right\}}{\sum_{i=0}^{m}\exp\left\{\sum_{k=1}^{p}f_k(a_i,l^*,l_i)\tilde{\beta}_k\right\}}
\end{aligned}
$$

$$
\begin{aligned}
&\qquad \times \frac{\exp\left\{\sum_{k=1}^{p} f_k(a_i, l^*, l_i)\beta_k\right\}}{\sum_{i=0}^{m} \exp\left\{\sum_{k=1}^{p} f_k(a_i, l^*, l_i)\beta_k\right\}}\Bigg] \\
&= \sum_{\{(l_0,a_0),\ldots,(l_m,a_M)\}} h(a_0, \ldots, a_M, l_0, \ldots, l_M) \\
&\qquad \times \sum_{u,i\in\{1,\ldots,m\}:i>u} [f_k(a_i, l^*, l_i) - f_k(a_u, l^*, l_u)] \\
&\qquad \times \Bigg[\frac{\exp\left\{\sum_{k=1}^{p} f_k(a_i, l^*, l_i)\tilde{\beta}_k\right\}}{\sum_{i=0}^{m} \exp\left\{\sum_{k=1}^{p} f_k(a_i, l^*, l_i)\tilde{\beta}_k\right\}} \frac{\exp\left\{\sum_{k=1}^{p} f_k(a_u, l^*, l_u)\beta_k\right\}}{\sum_{i=0}^{m} \exp\left\{\sum_{k=1}^{p} f_k(a_i, l^*, l_i)\beta_k\right\}} \\
&\qquad - \frac{\exp\left\{\sum_{k=1}^{p} f_k(a_u, l^*, l_u)\tilde{\beta}_k\right\}}{\sum_{i=0}^{m} \exp\left\{\sum_{k=1}^{p} f_k(a_i, l^*, l_i)\tilde{\beta}_k\right\}} \frac{\exp\left\{\sum_{k=1}^{p} f_k(a_i, l^*, l_i)\beta_k\right\}}{\sum_{i=0}^{m} \exp\left\{\sum_{k=1}^{p} f_k(a_i, l^*, l_i)\beta_k\right\}}\Bigg],
\end{aligned}
$$

which is clearly zero when $\tilde{\beta} = \beta$. If follows that

$$
\frac{\partial}{\partial \tilde{\beta}_k} f(\tilde{\beta}) = \mathbb{E}\left[\frac{\sum_{i=1}^{M} X_{i,k} g(X_i, \tilde{\beta})}{1 + \sum_{i=1}^{m} g(X_i, \tilde{\beta})}\middle| Y_K = 1, M > 0\right] = 0
$$

for all $k = 1, \ldots, p$ if and only if $\tilde{\beta} = \beta$. $\square$
